# Supplementary material for: Ferrostatin-1 and hinokitiol supplementation enhance human hematopoietic stem cell expansion in a chemically defined medium
Source: Mol Ther Adv. 2026 Mar 3;34(2):201711. doi: 10.1016/j.omta.2026.201711 (PMC13148910; doi:10.1016/j.omta.2026.201711)
Supplement: Document S1. Figures S1–S10 and Tables S1 and S2 [file mmc1.pdf]

OMTA, Volume *34*

Supplemental information

Ferrostatin-1 and hinokitiol supplementation enhance human hematopoietic stem cell expansion in a chemically defined medium

Lushen Li and Pankaj K. Mandal

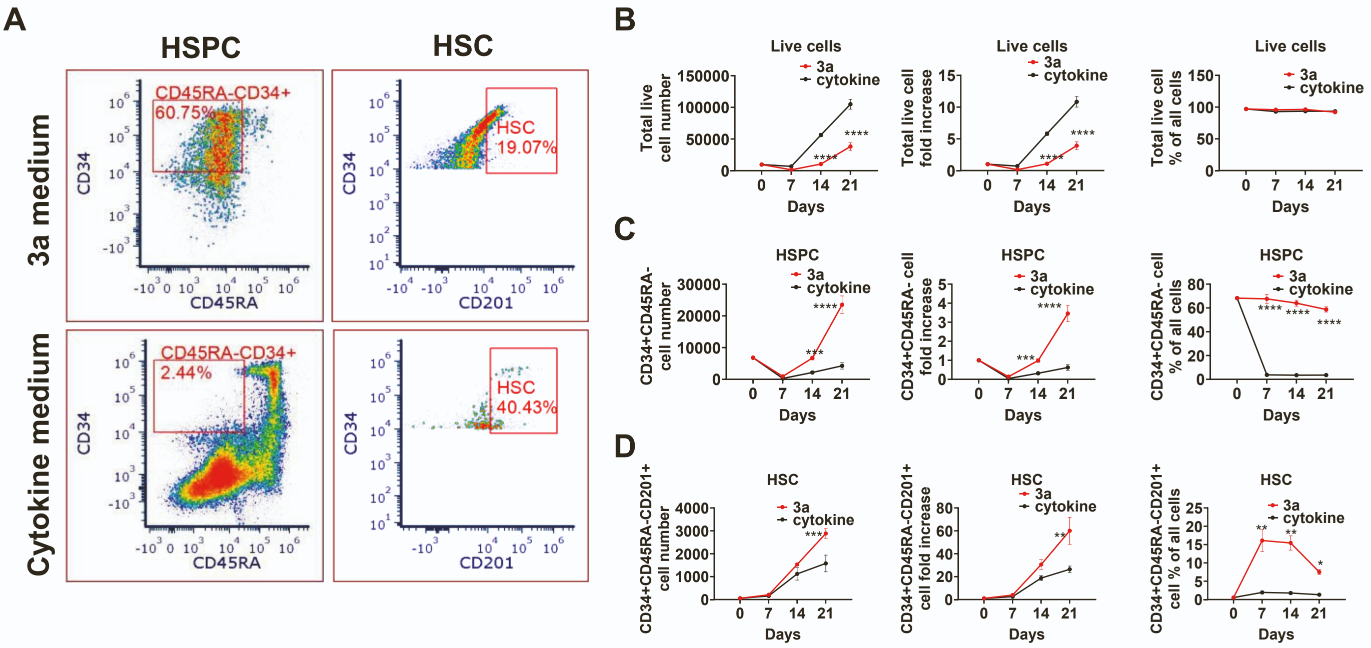

Figure S1: Human cord blood hematopoietic stem cell expansion in 3a medium. Human cord blood CD34+ cells were seeded in 96 well plates at 10,000 cells per well in 3a or cytokine-containing medium for 21 days. Immunophenotypic analyses were performed using flow cytometry. A: Gating strategy for HSPCs and HSCs analyses. B-D: Cell number (left), and fold increase (middle), and percentage of all cells (right) tracked over 21 days in 3a versus cytokine medium. B: Live cells, C: HSPCs, D: HSCs. Pooled data from two donors. Mean ± SEM. **P < 0.01, ***P < 0.001, ****P < 0.0001 by 2-way ANOVA with Šídák's multiple comparisons test.

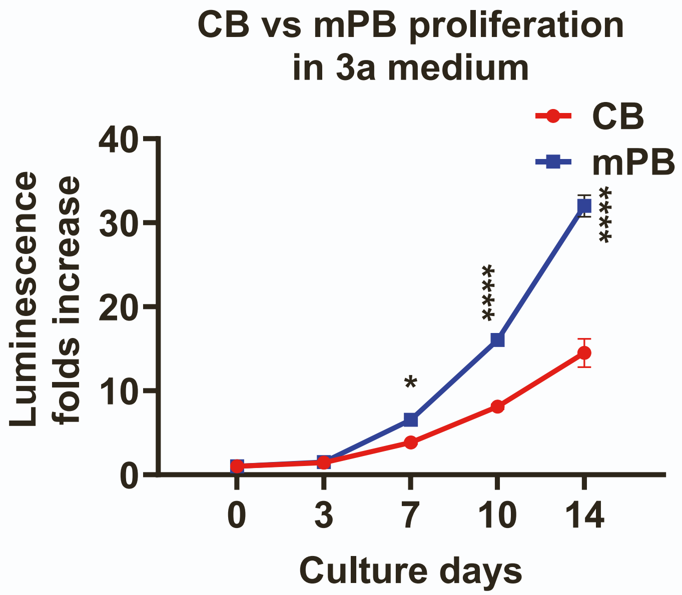

Figure S2: Cord blood vs peripheral blood cell proliferations in 3a medium. 25,000 human cord blood or peripheral blood-derived CD34+ cells were seeded in 96 well plate and cultured in 3a medium for 14 days. At each time point as indicated, cell proliferation was assessed using CellTiter-Glo® Luminescent Cell Viability Assay, data shows luminescence folds increase relative to the cells seeded at Day0. Data represents two times independent experiments. Data presented as mean ± SEM. *P < 0.05, ****P < 0.0001, by 2-way ANOVA with Šídák's multiple comparisons test.

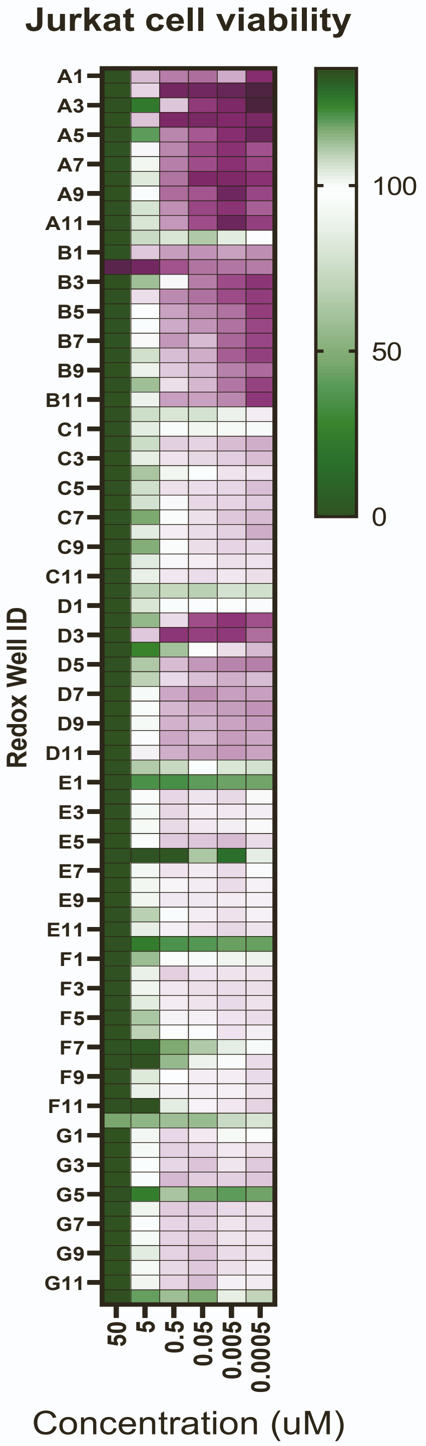

Figure S3: Titration of 83 antioxidant compound concentrations. Jurkat cells were seeded in 96 well plates at 10,000 cells per well in RPMI 1640 medium containing 1% Pen-Strep and 10% FBS. Cells were individually treated with 83 antioxidants at various concentrations ranged from 0.0005 to 50 uM. After 48 hours, cell proliferation was assessed using CellTiter-Glo® Luminescent cell viability assay, data shows luminescence folds relative to untreated cells. Data represents two times independent experiments.

A

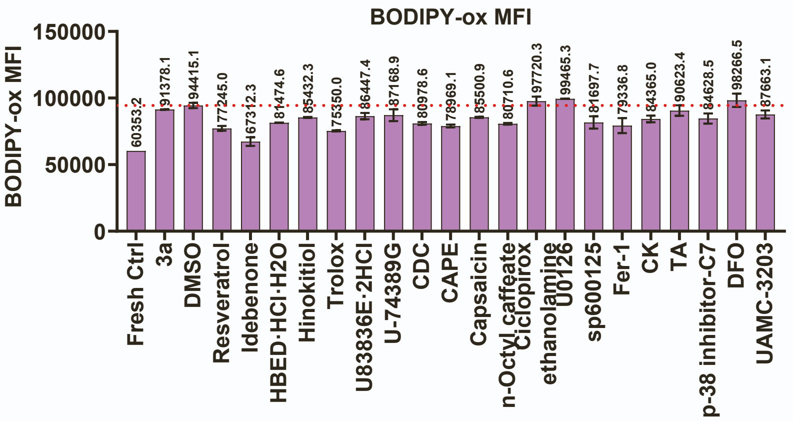

B

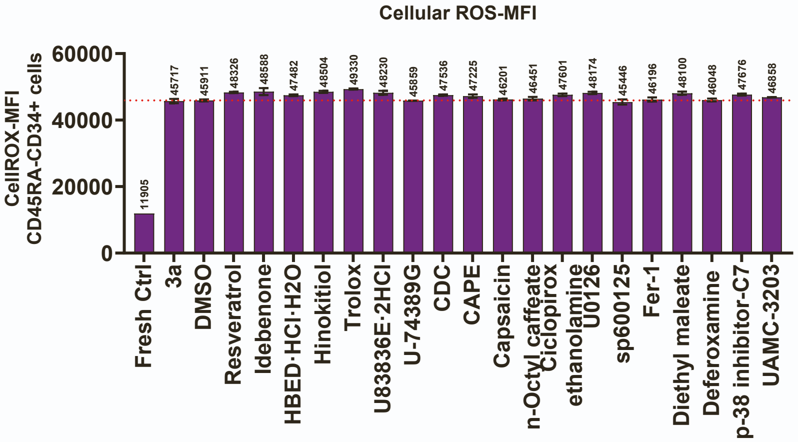

Figure S4: Lipid peroxidation and cellular ROS levels following treatment with selected compounds. Human CB cells were seeded in 96 well plates at 10,000 cells per well and cultured in 3a medium with selected compounds. A) Lipid peroxidation levels at day 14 analyzed by staining cells with BODIPY™ 581/591 C11. Data shows MFI in HSPCs. B) Cellular ROS levels at day 14 measured by staining cells with CellROX™ Deep Red. Data shows MFI in HSPCs. Representative data from three independent experiments are shown..

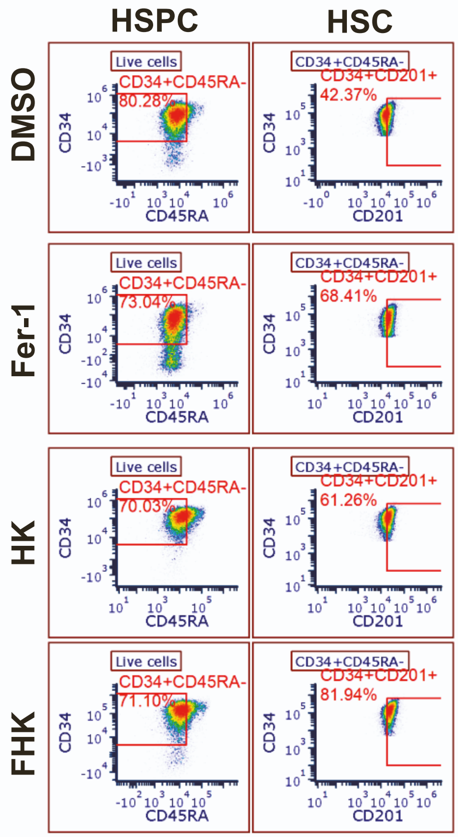

Figure S5: Representative FACS plots showing HSPC and HSC populations under each treatment conditions.

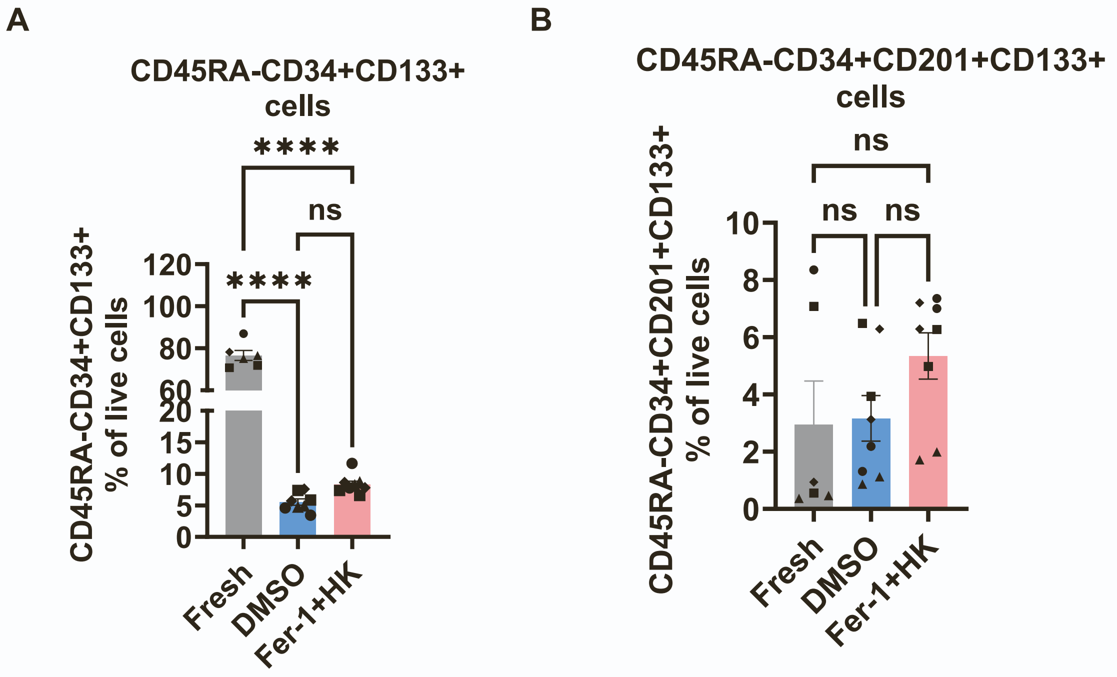

Figure S6: Immunophenotype analyses of *ex vivo* expanded CB cells in the presence of Fer-1 and Hinokitiol. Human CB cells were seeded in 6-well plates at 300,000 cells per well in 3a medium with DMSO (vehicle control), or Fer-1 (10uM) plus Hinokitiol (0.5uM) (FHK). A-B) Percentage of human CD45RA-CD34+CD133+ cells (A) and CD45RA-CD34+CD201+CD133+ (B) in live cells at day 14. Pooled data from three independent experiments with CD34+ cells from 4 donors (N=4) are presented as mean ± SEM. ****P < 0.0001 by 1-way ANOVA with Tukey's multiple-comparison test.

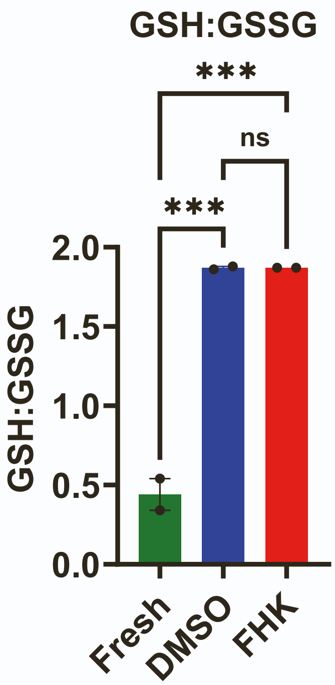

Figure S7: Effect of Hinokitiol and Fer-1 combination on human cord blood cell GSH:GSSG ratio. Human CB cells were cultured in 6-well plates at 300,000 cells per well and treated with DMSO (vehicle Ctrl), or Fer-1 plus Hinokitiol (FHK) for 14 days before GSH:GSSG ratio were measured following manufacture's instruction. Data presented as mean ± SEM by 1-way ANOVA with Tukey's multiple-comparison test. Pooled data from two donors are presented.

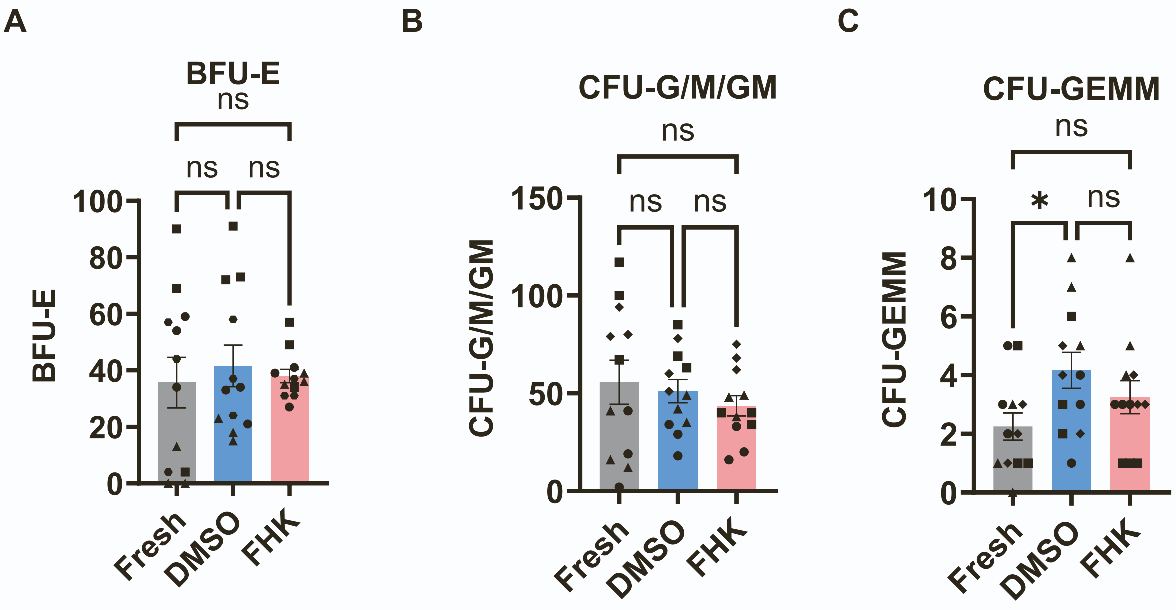

Figure S8: Effect of Hinokitiol and Fer-1 combination on human CB differentiation potential. Human CB cells were cultured in 6 well plates at 300,000 cells per well and treated with DMSO (vehicle Ctrl), or Fer-1 plus Hinokitiol (FHK) for 14 days. Then colony-forming unit (CFU) assay was initiated by seeding 1500 cultured cells per well in SmartDishTM 6-Well plates and culture at 37 °C with 5% $CO2$. After 14 days, plates were read with STEMvisionTM Automated CFU Assay Reader. Colony count of various types of colonies, including BFU-E (A), CFU-G/M/GM (B), and CFU-GEMN (C). Pooled data from three independent experiments with 4 donors showing colony count for various types. Data presented as mean ± SEM by 1-way ANOVA with Tukey's multiple-comparison test..

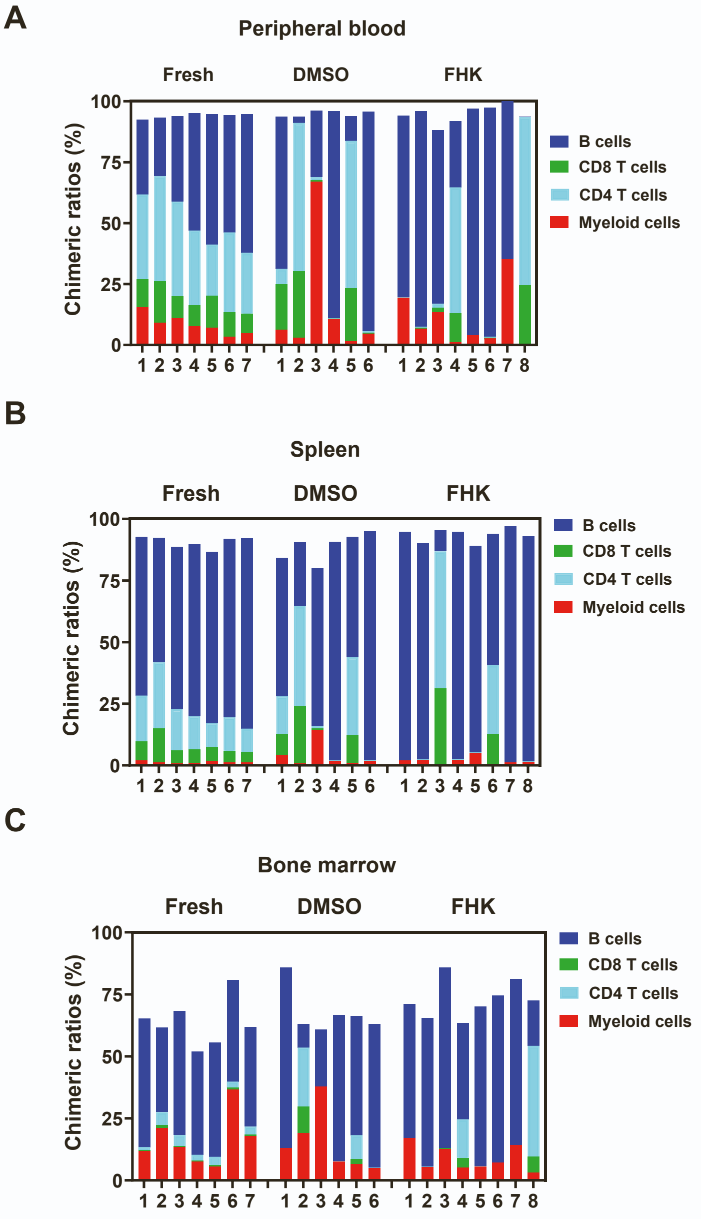

Figure S9: Lineage analyses of human blood cells in transplanted mice. Peripheral blood (A), spleen (B), and bone marrow (C) samples from individual mouse were analyzed at week 24 post-transplantation for lineage distribution of human blood cells by flow cytometry. X-axis showing number of mice in each group.

A

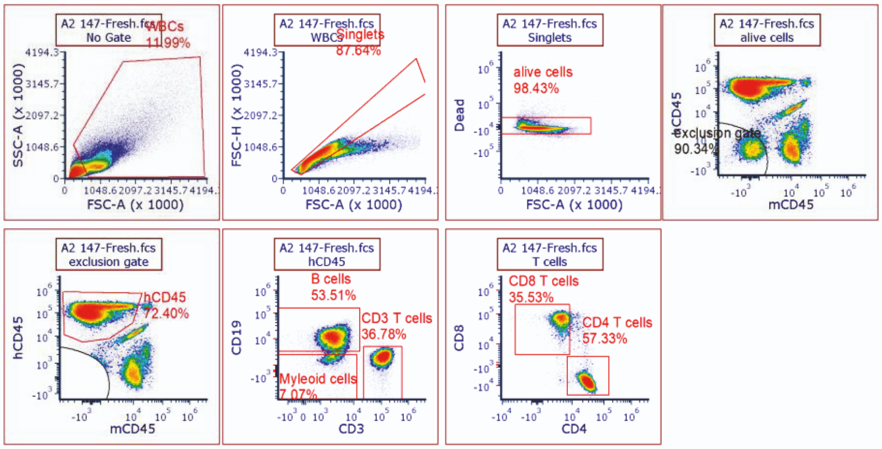

B

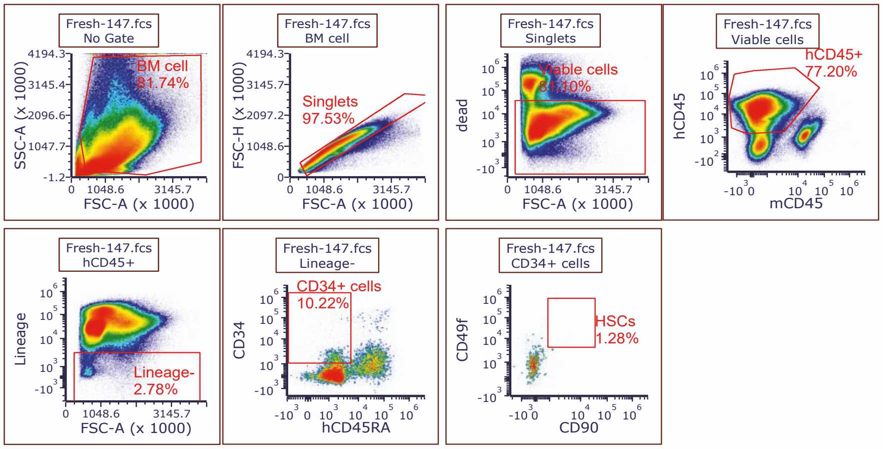

Figure S10: Representative FACS plots showing gating scheme for lineage (A) and HSPC (B) analyses.

Table S1: List of reagents.

Reagents	Clone	Flurophore	Vendor	Catalog
Alexa Fluor® 647 anti-human CD34	581	AF647	Biolegend	343508
BV421 Mouse Anti-Human CD34	581	BV421	BD Biosciences	562577
BV421 Mouse IgG1, k Isotype Control	X40	BV421	BD Biosciences	562438
Brilliant Violet 750™ anti-human CD45RA	HI100	BV750	Biolegend	304166
Brilliant Violet 750™ Mouse IgG1, κ isotype Ctrl	MOPC-21	BV750	Biolegend	400106
PE-labeled anti-human CD49c (ITGA3)	C3 II	PE	BD Biosciences	556025
APC anti-human CD133	clone 7	APC	Biolegend	372806
BV605 Rat Anti-Human CD201	RCR-252	BV605	BD Biosciences	743553
Brilliant Violet 421™ anti-human CD90 (Thy1)	5E10	BV421	Biolegend	328122
Brilliant Violet 785™ anti-human CD38	HIT2	BV785	Biolegend	303530
BV605 Rat IgG1, k Isotype Control	R3-34	BV605	BD Biosciences	562993
FITC Human lineage cocktail 4	--	FITC	BD Biosciences	562722
Zombie yellow viability kit.	---	---	Biolegend	423103
Zombie Aqua™ Fixable Viability Kit	---	---	Biolegend	423102
Human TruStain FcX™ (Fc Receptor Blocking Solution)	--	--	Biolegend	422302
Cell Staining Buffer	---	---	Biolegend	420201
Compensation Beads	---	---	Biolegend	424602

Table S2: List of compounds in Screen-Well REDOX Library (BML-2835 Version 1.4).

No.	Plate Location	Cat. No.	Compound	CAS #	MW	Conc.	Solvent	Activity
1	A-01	AC765	Promethazine·HCl	58-33-3	320.9	10mM	DMSO	Secondary anti-oxidant
2	A-02	RD100	Cumene hydroperoxide	80-15-9	152.2	10mM	DMSO	Aryl hydroperoxide
3	A-03	GR308	β-Lapachone	4707-32-8	242.3	10mM	DMSO	Undergoes futile redox cycles
4	A-04	FR104	Resveratrol	501-36-0	228.2	10mM	DMSO	Stilbene phenolic antioxidant
5	A-05	RD101	Hydroquinone	123-31-9	110.1	10mM	DMSO	Phenolic antioxidant
6	A-06	FR118	TEMPOL	2226-96-2	172.2	10mM	DMSO	SOD mimetic
7	A-07	FR116	Ferulic acid ethylester	4046-02-0	222.2	10mM	DMSO	Phenolic antioxidant
8	A-08	FR115	D-α-Tocopherylquinone	7559-04-8	446.7	10mM	DMSO	Oxidized vitamin E
9	A-09	RA119	Seratrodast	112665-43-7	354.4	10mM	DMSO	Quinone antioxidant
10	A-10	FR114	Idebenone	58186-27-9	338.4	10mM	DMSO	Quinone antioxidant
11	A-11	270061	t-Butylhydroquinone	1948-33-0	166.2	10 mM	DMSO	Phenolic antioxidant
12	A-12	FR113	HBED·HCl·H2O	35369-53-0 (base molecule)	442.9	10 mM	DMSO	Metal chelator
13	B-01	FR112	Ambroxol	18683-91-5	378.1	10mM	DMSO	Nonphenolic antioxidant
14	B-02	FR111	L-Ergothioneine	497-30-3	229.3	10mM	H2O	Endogenous antioxidant
15	B-03	FR110	Hinokitiol	499-44-5	164.2	10mM	DMSO	Metal chelator
16	B-04	270263	(-)-Epigallocatechin gallate	989-51-5	458.4	10 mM	DMSO	Polyphenol
17	B-05	FR106	Procysteine	19771-63-2	147.2	10mM	DMSO	Glutathione precursor
18	B-06	FR105	Trolox	53188-07-1	250.3	10mM	DMSO	Short-chain vitamin E analog
19	B-07	FR103	MCI-186	89-25-8	174.2	10mM	DMSO	Nonphenolic antioxidant
20	B-08	FR101	U83836E·2HCl	137018-55-4	593.6	10mM	DMSO	Antioxidant Lazaroid
21	B-09	FR100	U-74389G	153190-29-5	726.9	10 mM	DMSO	Antioxidant Lazaroid
22	B-10	EI363	GERI-BP002A	119-47-1	340.5	10mM	DMSO	Phenolic antioxidant
23	B-11	EI345	Apigenin	520-36-5	270.2	10mM	DMSO	Flavone antioxidant
24	B-12	EI318	Terbinafine·HCl	78628-80-5	327.9	10mM	DMSO	Free radical quencher
25	C-01	270253	Rosmarinic acid	537-15-5, 20283-92-5	360.3	10 mM	DMSO	Phenolic antioxidant
26	C-02	270202	Piceatannol	10083-24-6	244.2	10 mM	DMSO	Stilbene phenolic antioxidant
27	C-03		Blank					

28	C-04	EI211	CDC	132465-11-3	321.3	10mM	DMSO	Phenolic antioxidant
29	C-05	EI207	Ebselen	60940-34-3	274.2	10mM	DMSO	Glutathione peroxidase mimetic
30	C-06	350006	Genistein	446-72-0	270.2	10mM	DMSO	Isoflavone antioxidant
31	C-07	EI135	Curcumin	458-37-7	368.4	10mM	DMSO	Phenolic antioxidant
32	C-08	EI134	Phenidone	92-43-3	162.2	10mM	DMSO	Nonphenolic antioxidant
33	C-09	EI130	Gossypol	303-45-7	518.6	10mM	DMSO	Phenolic antioxidant
34	C-10	EI129	Gentisic acid	490-79-9	154.1	10mM	DMSO	Phenolic antioxidant
35	C-11	EI124	Caffeic acid	331-39-5	180.2	10mM	DMSO	Phenolic antioxidant
36	C-12	EI106	Baicalein	491-67-8	270.2	10mM	DMSO	Flavone antioxidant
37	D-01	EI100	Esculetin	305-01-1	178.1	10mM	DMSO	Coumarin antioxidant
38	D-02	RD103	Propyl gallate	121-79-9	212.2	10mM	DMSO	Phenolic antioxidant
39	D-03	ET004	5,8,11,14-Eicosatetraynoic acid	1191-85-1	296.4	10mM	DMSO	Acetylenic antioxidant
40	D-04	FR102	CAPE	104594-70-9	284.3	10mM	DMSO	Phenolic antioxidant
41	D-05	EI101	Nordihydroguaiaretic acid	500-38-9	302.4	10mM	DMSO	Phenolic antioxidant
42	D-06	EI125	Capsaicin	404-86-4	305.4	10mM	DMSO	Phenolic antioxidant
43	D-07	RD104	BHT	128-37-0	220.4	10mM	DMSO	Phenolic antioxidant
44	D-08	RD105	BHA	121-00-6	180.2	10mM	DMSO	Phenolic antioxidant
45	D-09	350144	Bakuchiol	10309-37-2	256.4	10mM	DMSO	Phenolic antioxidant
46	D-10	RD106	DL-α-Lipoic acid	1077-28-7	206.3	10mM	DMSO	Sulfur-containing antioxidant
47	D-11	RD107	Eugenol	97-53-0	164.2	10mM	DMSO	Phenolic antioxidant
48	D-12	NS520	Melatonin	73-31-4	232.3	10mM	DMSO	Nonphenolic antioxidant
49	E-01	105005	N-Acetyl-Cysteine	616-91-1	163.2	10mM	DMSO	Thiol-containing reducing agent
50	E-02	RD109	D-γ-Tocopherol	54-28-4	416.7	10mM	DMSO	Phenolic antioxidant
51	E-03	AP308	Tocopherol succinate	4345-03-3	530.8	10mM	DMSO	Phenolic antioxidant
52	E-04	RD110	Ascorbic acid	50-81-7	176.1	10mM	DMSO	Ascorbate-type antioxidant
53	E-05	RD111	Ascorbyl palmitate	137-66-6	414.5	10mM	DMSO	Lipohilic ascorbate
54	E-06	RD112	n-Octyl caffeate	NA	292.4	10mM	DMSO	Phenolic antioxidant
55	E-07	RD113	Paeonol	552-41-0	166.2	10mM	DMSO	Phenolic antioxidant
56	E-08	RD129	Protocatechuic acid	99-50-3	154.1	10mM	DMSO	Phenolic antioxidant
57	E-09	RD114	Glutathione	70-18-8	307.3	10mM	DMSO	Thiol-containing reducing agent

58	E-10	AR112	Carvedilol	72956-09-3	406.5	10mM	DMSO	Nonphenolic antioxidant
59	E-11	RD115	Diludine	1149-23-1	253.3	10 mM	DMSO	Dihydropyridine-type antioxidant
60	E-12	270264	Carnosic acid	3650-09-7	332.4	10mM	DMSO	Phenolic antioxidant
61	F-01	GR336	Tanshinone IIA	568-72-9	294.3	10mM	DMSO	1,2-Quinone antioxidant
62	F-02	RD116	Probucol	23288-49-5	516.8	10mM	DMSO	Phenolic antioxidant
63	F-03	FA001	Eicosapentaenoic acid	10417-94-4	302.5	10 mM	DMSO	Polyunsaturated radical scavenger
64	F-04	FA002	Docosa-4Z,7Z,10Z,13Z,16Z,19Z-hexaenoic acid	6217-54-5	328.5	10mM	DMSO	Polyunsaturated radical scavenger
65	F-05	NP576	Bis demethoxycurcumin	24939-16-0	308.3	10 mM	DMSO	Phenolic antioxidant
66	F-06	EI375	Ibuproxam	53648-05-8	221.3	10mM	DMSO	Metal chelator
67	F-07	RD128	Ciclopirox ethanolamine	41621-49-2	268.4	10mM	DMSO	Hydroxyl radical scavenger
68	F-08	NP536	Thymoquinone	490-91-5	164.2	10mM	DMSO	Quinone antioxidant
69	F-09	RD117	Thiourea	62-56-6	76.1	10mM	DMSO	Thiol-containing reducing agent
70	F-10	280001	DTT	3483-12-3	154.3	10 mM	DMSO	Thiol-containing reducing agent
71	F-11	PI149	N-Ethylmaleimide	128-53-0	125.1	10mM	DMSO	Thiol trap
72	F-12	FR117	L-Buthionine-sulfoximine	83730-53-4	222.3	10 mM	water	Glutathione biosynthesis inhibitor
73	G-01	DL151	Anethole trithione	532-11-6	240.4	10mM	DMSO	Sulfur-containing antioxidant
74	G-02	RD119	TEMPO	2564-83-2	156.2	10mM	DMSO	Nitroxyl radical
75	G-03	ST330	D609	83373-60-8	266.5	10mM	DMSO	Antioxidant
76	G-04	270212	Captopril	62571-86-2	217.3	10 mM	DMSO	Thiol-containing reducing agent
77	G-05	DL379	Disulfiram	97-77-8	296.5	10 mM	DMSO	Sulfur-containing antioxidant
78	G-06	RD121	1,2-Dithiole-3-thione	534-25-8	134.2	10 mM	DMSO	Sulfur-containing antioxidant
79	G-07	RD122	Selenomethionine	3211-76-5	196.1	10mM	DMSO	Selenium-containing antioxidant
80	G-08	RD123	Tetramethylpyrazine	1124-11-4	136.2	10mM	DMSO	Nonphenolic antioxidant
81	G-09	RD124	Ethoxyquin	91-53-2	217.3	10mM	DMSO	Nonphenolic antioxidant
82	G-10	RD125	Canthaxanthin	514-78-3	564.8	1mM	DMSO	Polyunsaturated radical scavenger
83	G-11	RD126	β-carotene	7235-40-7	536.9	10mM	DMSO	Polyunsaturated radical scavenger
84	G-12	RD127	Retinyl palmitate	79-81-2	524.9	10mM	DMSO	Polyunsaturated radical scavenger
