# Supplementary material for: Ferrostatin-1 and hinokitiol supplementation enhance human hematopoietic stem cell expansion in a chemically defined medium
Source: Mol Ther Adv. 2026 Mar 3;34(2):201711. doi: 10.1016/j.omta.2026.201711 (PMC13148910; doi:10.1016/j.omta.2026.201711)
Supplement: Document S2. Article plus supplemental information [file mmc2.pdf]

Molecular Therapy
Advances
Original Article

Ferrostatin-1 and hinokitiol supplementation enhance human hematopoietic stem cell expansion in a chemically defined medium

Lushen Li[1] and Pankaj K. Mandal[1]

[1]Tumor Vaccine and Biotechnology Branch, Division of Cell Therapy 2, Office of Cellular Therapy and Human Tissue, Office of Therapeutic Products, Center for Biologics Evaluation and Research, Food and Drug Administration, Silver Spring, MD, USA

An optimized and standardized method for *ex vivo* expansion of cord blood (CB) hematopoietic stem cells (HSCs) in a chemically defined medium has yet to be established. In this study, we aimed to improve *ex vivo* expansion of HSCs in a recently developed cytokine-free, chemically defined 3a medium. We found that the co-supplementation of the ferroptosis inhibitor ferrostatin-1 and iron chelator hinokitiol (FHK) in 3a medium significantly improves CB HSC expansion by suppressing lipid peroxidation and mitigating oxidative stress. FHK supplementation improves overall cell proliferation and promotes preferential expansion of HSCs without adversely affecting the clonogenic, engraftment, and differentiation potential of HSCs. The delayed engraftment kinetics with gradual increase in hematopoietic output further suggests that FHK treatment may preserve or expand long-term HSCs. Our findings are in alignment with recent published studies highlighting the susceptibility of HSCs to ferroptosis and corroborate the use of antioxidants to improve *ex vivo* expansion of HSC.

INTRODUCTION

Cord blood (CB) $CD34^+$ cells are an excellent source of allogeneic hematopoietic stem and progenitor cells (HSCs/HSPCs) for hematopoietic stem cell transplantation (HSCT) to treat various blood disorders. However, the limited number of HSCs poses a significant challenge for HSC research and their wider clinical applications. Although roughly 800,000 cord units are available for transplantation in public CB banks (https://wmda.info), only 4%–5% of available cord units have adequate $CD34^+$ cells to meet the HSCT dose requirement, particularly for adult patients.[1] A retrospective analysis showed that *ex vivo* expansion of CB $CD34^+$ cells with UM171 could significantly increase donor availability compared to single and double unmanipulated CB transplantation, even for ethnic minorities for whom finding a compatible donor is challenging.[1] This underscores the need to expand CB $CD34^+$ cells *ex vivo* to address unmet medical need by increasing access to HSCT for a significant number of patients. Decades of research in this space led to the identification of a number of *ex vivo* expansion strategies, such as tetraethylenepentamine (TEPA),[2,3] nicotinamide,[4–6] delta-1 ligand,[7,8] StemRegenin-1(SR-1),[9,10] UM171,[11–13] resveratrol,[14] prostaglandin(dm-PEG2),[15,16] MAP kinase inhibitor C7,[17] JNK inhibitor JNK-IN-8,[18,19] and others.[20,21] These strategies employed serum/human albumin and included cytokines for supporting *ex vivo* proliferation of HSCs. Despite intensive research, a standardized optimal medium that robustly supports *ex vivo* expansion and maintenance of human CB HSCs has yet to be established. Moreover, from quality and control perspective, use of cytokines and animal/human-derived components in commonly used media for *ex vivo* expansion of HSCs poses significant challenges and limitations, such as difficulty in determining the optimal concentration and precise combinations, short half-lives, batch-to-batch variations, high prices, functional redundancies, immunogenicity, and side effects.[22–24] In addition, the use of animal/human-derived components also poses significant safety risk via the introduction of adventitious viruses and human pathogens. These challenges further necessitate additional optimization to move toward a chemically defined medium for HSC/HSPC expansion. Toward this end, a recent study described a chemically defined, cytokine- and albumin-free culture medium known as “3a medium” by substituting exogenous cytokines and albumin with chemical agonists and a caprolactam-based polymer.[25] This medium effectively supports long-term *ex vivo* expansion of human CB HSC/HSPCs. The 3a medium can enhance batch-to-batch consistency, reduce costs, and support rapid clinical translation.

However, Sakurai et al. reported that $CD34^+$ cells cultured in 3a medium showed higher levels of reactive oxygen species (ROS), increased membrane lipid peroxidation, and γH2AX accumulation as compared to fresh cells.[25] Physiological levels of ROS play a critical role in regulating HSC development, self-renewal, migration, and differentiation through the modulation of cell signaling pathways.[26,27] However, elevated ROS levels and resulting oxidative stress are detrimental to HSPC functions,[26,28–31] may induce or affect cellular differentiation,[32,33] and could be an important pathogenic factor in hematological diseases.[34–36] Elevated ROS resulted in loss

Received 14 August 2025; accepted 26 February 2026;
https://doi.org/10.1016/j.omta.2026.201711.

Correspondence: Pankaj K. Mandal, Tumor Vaccine and Biotechnology Branch, Division of Cell Therapy 2, Office of Cellular Therapy and Human Tissue, Office of Therapeutic Products, Center for Biologics Evaluation and Research, Food and Drug Administration, Silver Spring, MD, USA.
E-mail: pankaj.mandal@fda.hhs.gov

Published by Elsevier Inc. on behalf of The American Society of Gene and Cell Therapy.
This is an open access article under the CC BY license (http://creativecommons.org/licenses/by/4.0/).

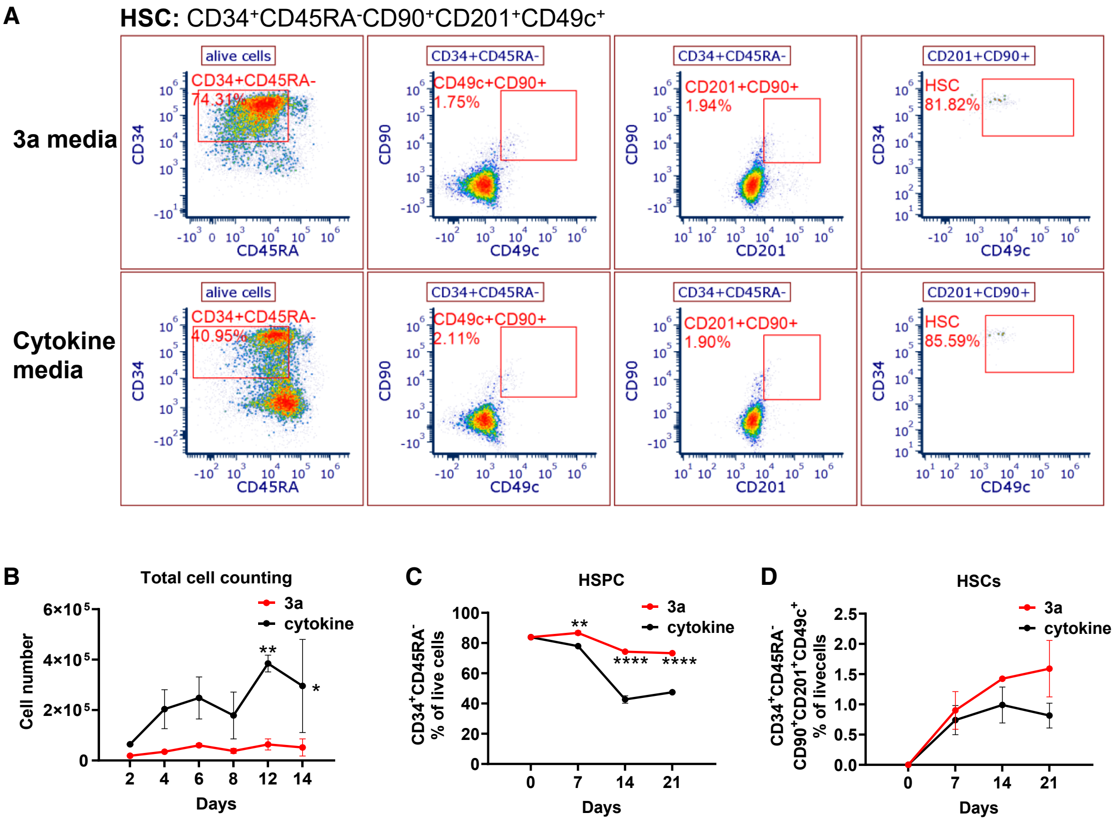

Figure 1. 3a medium preserves hematopoietic stem cell immunophenotype better than cytokine medium

Human CB cells were seeded in 96-well plates at 10,000 cells per well in 3a or cytokine medium. Cell phenotype was analyzed using flow cytometry. (A) Gating scheme used for immunophenotypic HSPC and HSC analyses in the CB cells cultured for 14 days. (B) Total cell number tracked for 14 days' culture. (C and D) Immunophenotypic HSPC (C) and HSC (D) percentages in live cells at indicated time points. Data represent three independent experiments, each performed with unique CB donor in duplicate. Mean ± SD. *$p < 0.05$, **$p < 0.01$, ****$p < 0.0001$ by two-way ANOVA with Sidak's multiple comparisons test.

in HSC quiescence and reduced HSC number. Restoration to lower ROS levels resulted in high reconstitution capacity.[26] Therefore, we hypothesized that antioxidant supplementation may improve HSC/HSPCs expansion in 3a medium. In this study, we screened 84 antioxidants, and 7 chemical inhibitors linked to ROS-regulated signaling pathways and found that co-supplementation of ferrostatin-1 (Fer-1) and the iron chelator hinokitiol (HK) enabled preferential expansion of CB HSCs by suppressing lipid peroxidation and ameliorating oxidative stress.

RESULTS

Chemically defined 3a medium allows expansion of CD34$^+$ HSPCs

To confirm that chemically defined 3a medium is supportive of CD34$^+$ HSC/HSPC expansion, we first cultured CB CD34$^+$ cells in 3a medium for 3 weeks. For comparison, we also cultivated CB CD34$^+$ cells from the same donor in a cytokine-containing medium (TPO: 100 ng/mL; SCF: 10 ng/mL). The CD34$^+$ cells were immunophenotyped (Figures 1A, 1C, and 1D) on day 7, 14, and 21 and counted (Figure 1B) every other day of *ex vivo* culture. Though the total cell yield was significantly lower in 3a medium (Figure 1B), the percentage of CD34$^+$CD45RA$^-$ cells were higher in 3a medium compared to cytokine-containing medium (Figures 1A and 1C). When we looked for immunophenotypically defined HSCs (characterized by CD34$^+$CD45RA$^-$CD90$^+$CD201$^+$CD49c$^+$) in the *ex vivo* expanded cells, we found that only 2% of CD34$^+$CD45RA$^-$ cells were positive for HSCs marker expression (Figures 1A and 1D) which was comparable to HSC in cytokine-containing medium. These data suggest that although the overall cell proliferation is less in 3a medium compared to cytokine-containing medium, *ex vivo* expanded cells in 3a medium contained higher proportion of HSPC and immunophenotypically defined HSC. We performed additional experiments to determine if there was an increase in absolute number of HSPCs and HSCs during 21 days of *ex vivo* culture. We plated 10,000 freshly thawed CD34$^+$ cells/well in a 96-well plate and cultured them for 21 days. Expanded cells were analyzed by flow cytometry at day 7, 14, and 21 of culture by acquiring all the cells present in a well to determine the absolute number of total cells. Immunophenotype data were used to compute the total number of HSPCs and HSCs. We observed higher increase in total cell number in cytokine-containing medium compared to 3a

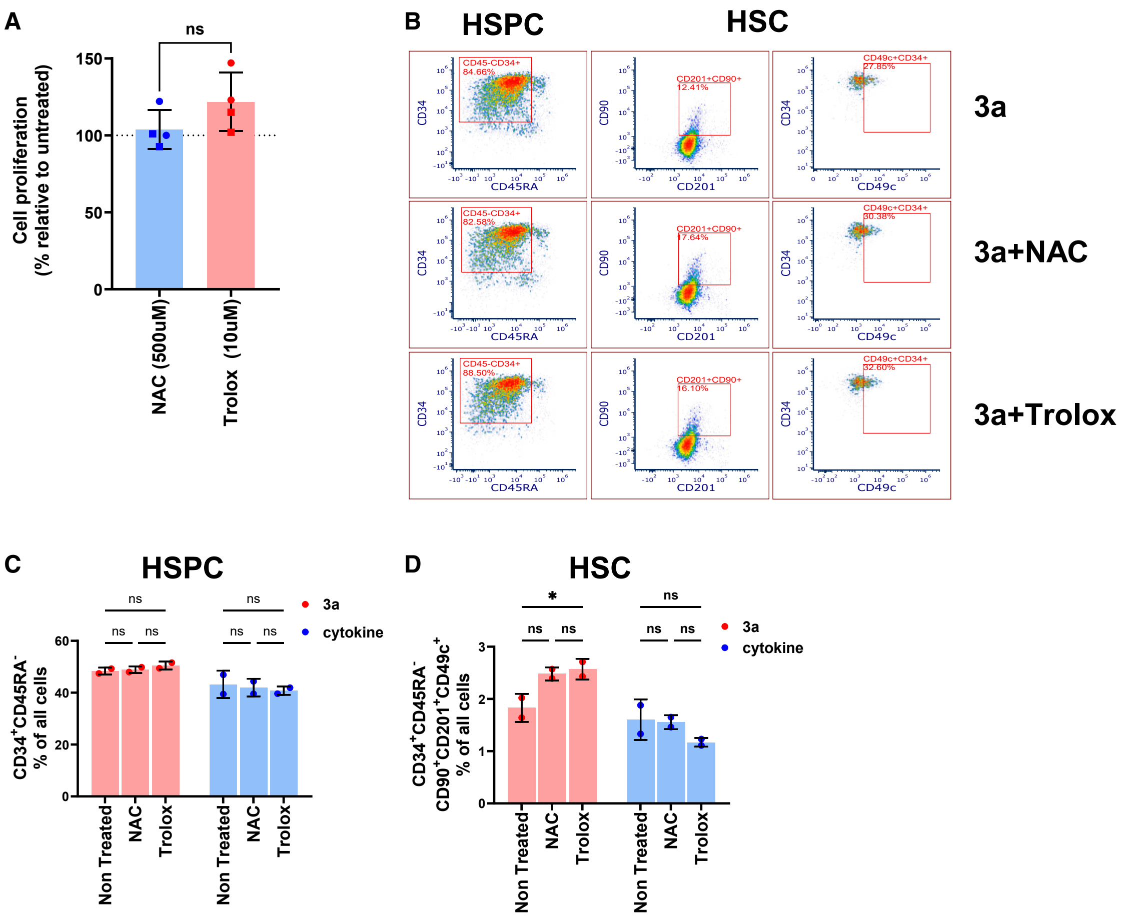

Figure 2. Antioxidants preserved HSC immunophenotype

Human CB cells were seeded in 96 well plates at 10,000 cells per well in 3a or cytokine medium. Cells were treated with N-acetylcysteine (NAC, 500 μM) or trolox (10 μM). (A) Cell proliferation assessed using CellTiter-Glo Luminescent cell viability assay. Pooled data from two experiments, each performed with unique CB donor (represented by ■ and ●) in duplicate. Mean ± SEM, *t* tests with Mann-Whitney test. (B) Representative FACS plots showing gating scheme for immunophenotypic analyses of HSPCs and HSCs. (C and D) After 14 days' culture, immunophenotypic HSPC (C) and HSC (D) percentages were analyzed using flow cytometry. Representative data from two independent experiments, each performed with unique CB donor in duplicate. Mean ± SD. $^{*}p < 0.05$ by two-way ANOVA with Tukey's multiple-comparison test.

medium (Figure S1B). However, absolute HSPC and HSC number were significantly higher in 3a medium compared to cytokine-containing medium (Figures S1A, S1C, and S1D).

We then tested if 3a medium is also supportive of mobilized peripheral blood (mPB) $CD34^+$ cell expansion. For this purpose, we compared the proliferation of CB and mPB $CD34^+$ cells in 3a medium. After 2 weeks of *ex vivo* culture, we observed 32-fold expansion of mPB $CD34^+$ cells compared to 14-fold expansion of CB $CD34^+$ cells (Figure S2). Taken together, these results indicate that 3a medium is supportive of $CD34^+$ cell expansion.

Antioxidant supplementation improves $CD34^+$ cell expansion in 3a medium

Given that there was an increased ROS accumulation and lipid peroxidation in 3a medium, we hypothesized that antioxidant supplementation might improve HSPC/HSC expansion in 3a medium. To test our hypothesis, we started with two antioxidants N-acetylcysteine (NAC) and trolox (a water-soluble analog of vitamin E) that are well-known for their strong antioxidant and cytoprotective activity from oxidative stress.[37–42] We found that though there was no significant increase in cell proliferation/yield (Figure 2A) or total HSPCs (Figure 2C), trolox supplementation led to a slight but significant increase in the number of HSCs ($CD34^{-}$ $^{+}CD45RA^{-}CD90^{+}CD201^{+}CD49c^{+}$ cells) in 3a medium compared to non-treated control group (Figures 2B and 2D). In contrast, neither antioxidant was effective in increasing the number of HSPC or HSC in cytokine-containing medium (Figures 2C and 2D).

Screening antioxidant compounds for HSC expansion

Promising results with two test antioxidants (Figure 2), in particular trolox, encouraged us to conduct a chemical library screening to

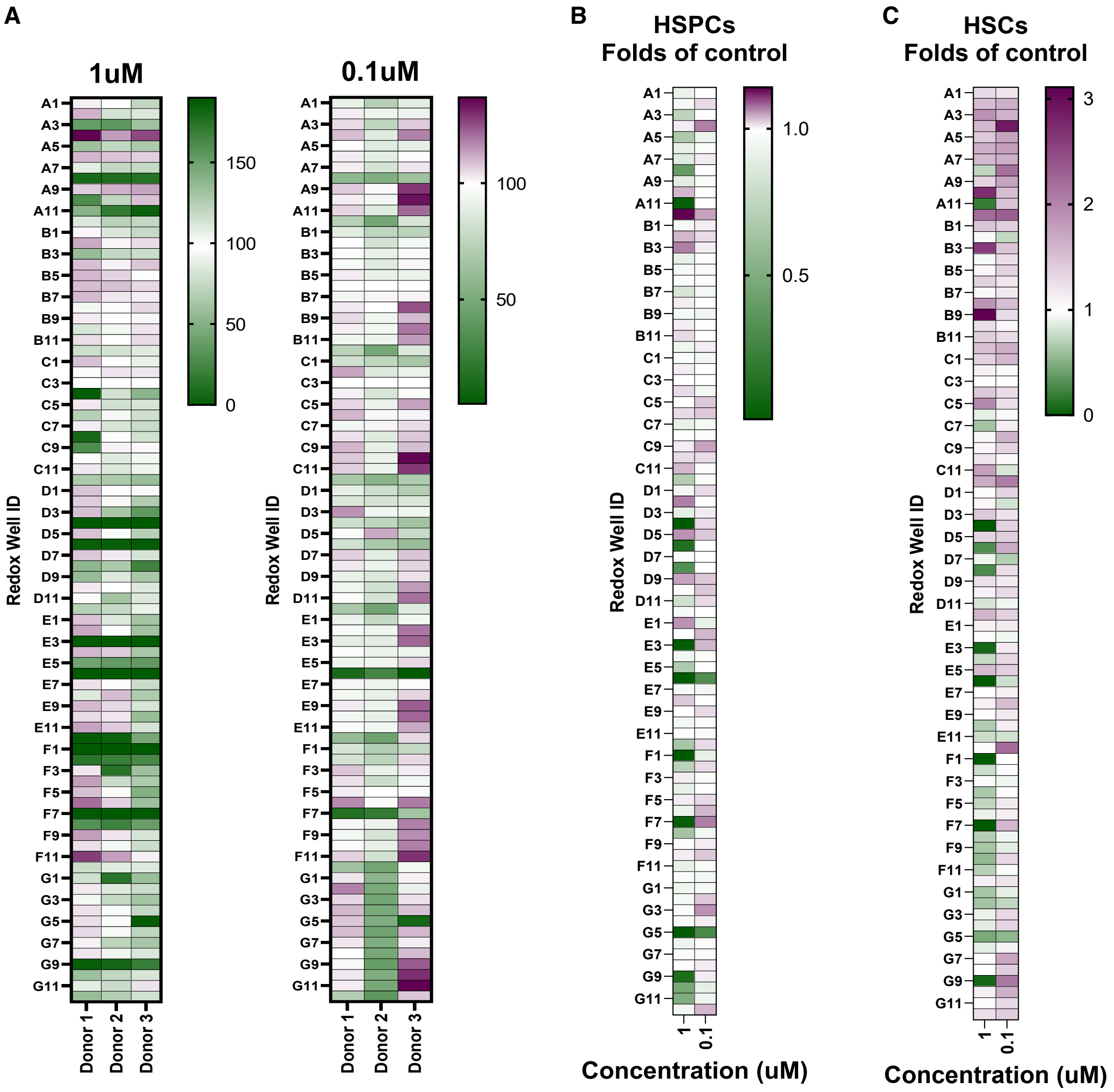

Figure 3. Effect of antioxidant compounds on human CB CD34⁺ cell expansion and immunophenotype

(A) Relative proliferation of CB $CD34^+$ cells at day 14 in 3a medium supplemented with each of the compounds in SCREEN-WELL REDOX library. Data from three independent experiments, each performed with unique CB donor, are represented as relative luminescence (%) to DMSO-treated cells. Each row represents a compound. Well ID is shown on the left. (B and C) Fold change in HSPC (B) and HSC (C) fraction under each treatment condition relative to DMSO control group at day 14 of *ex vivo* culture. Representative data from 3 independent experiments, each performed with unique CB donor.

identify additional antioxidants that could improve HSC expansion/maintenance in 3a medium. For this purpose, we used the SCREEN-WELL REDOX library (Enzo, Catalog # BML-2835) which contains 83 compounds with defined antioxidant or prooxidant activity (Table S2). To identify a non-toxic working concentration for each of these compounds, we performed a dose-titration experiment with Jurkat cells by testing serial log-10 diluted concentrations of each of these compounds starting with 50 μM. As shown in Figure S3, we observed an inverse dose-dependent response on the cell viability. Based on dose titration data, we decided to test 1 μM and 0.1 μM concentrations of each of these compounds on CB $CD34^+$ cells. CB $CD34^+$ cells from three independent donors were cultured for 2 weeks in the presence of each of these compounds at 1 μM and 0.1 μM in 96-well plate. Cell proliferation (Figure 3A) and immunophenotype (Figures 3B and 3C) analyses were carried out on day 14 of the *ex vivo* culture. From this screening, we were able to identify some of the compounds (such as resveratrol) know to improve *ex vivo* expansion of CB $CD34^+$ cells.[14] Not surprisingly, we also found that prooxidants (such as L-buthionine sulfoximine (BSO), well ID F12, Table S2) adversely affected CB $CD34^+$ cell proliferation (Figure 3A). We identified 12 compounds (resveratrol, idebenone, HBED·HCl·H_2O, hinokitiol, trolox, U83836E·2HCl,

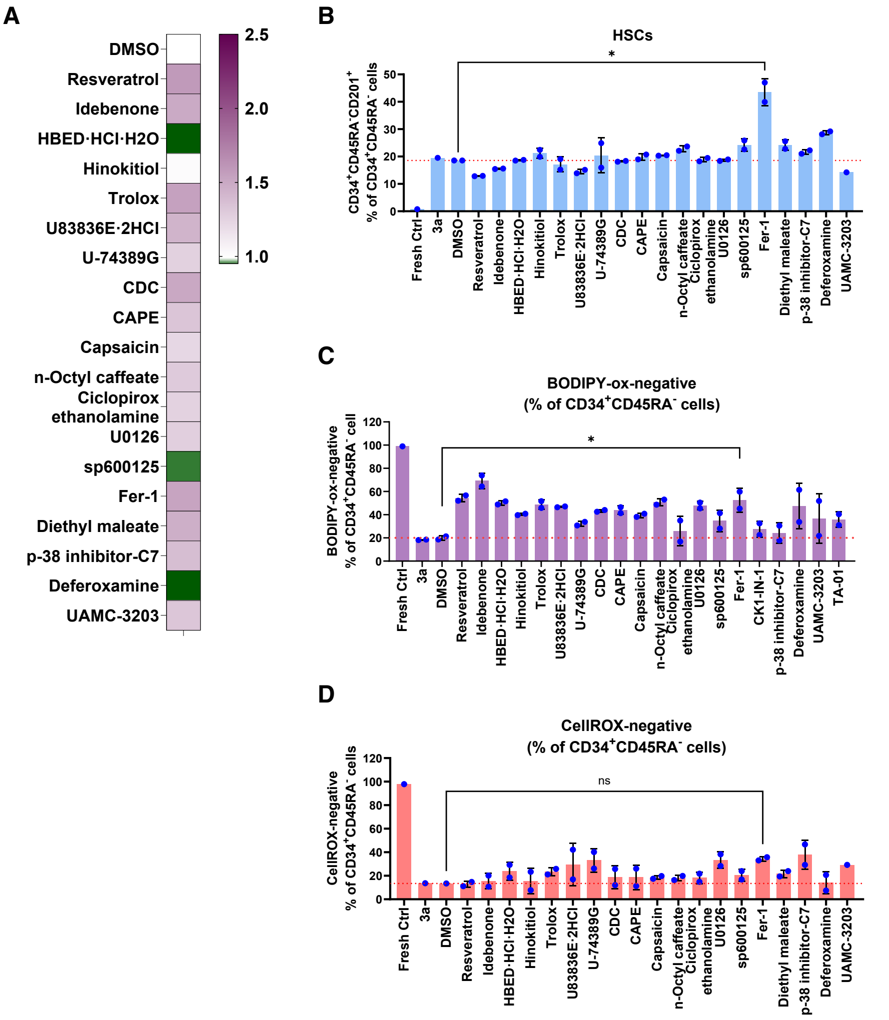

Figure 4. Effect of selected compounds on CD34$^+$ cell expansion in 3a medium

Human CB CD34$^+$ cells were seeded in 96-well plates at 10,000 cells per well in 3a medium with selected compounds. (A) Relative cell proliferation (fold increase) in the presence of selected compounds at day 14 compared to untreated control cells. Cell proliferation was assessed using CellTiter-Glo Luminescent cell viability assay. (B) Immunophenotypic analysis showing HSC percentage within HSPCs. (C) Lipid peroxidation levels at day 14 following treatment with selected compounds analyzed by BODIPY 581/591 C11 staining. Data show BODIPY-ox negative portion (%) in HSPCs. (D) Cellular ROS levels at day 14 following treatment with selected compounds measured with CellROX Deep Red. Data show negative portion (%) in HSPCs. Representative data from three independent experiments, each performed with unique CB donor in duplicate, are shown. (B–D) Mean ± SD, statistical analyses were conducted between Fer-1 versus DMSO vehicle control by *t* test, **p* < 0.05.

U-74389G, CDC, CAPE, capsaicin, n-octyl caffeate, and ciclopirox ethanolamine) that improved expansion of CB CD34$^+$ cells and/or maintenance of HSPC (CD34$^+$CD45RA$^-$) or HSC phenotype (CD34$^+$CD45RA$^-$CD201$^+$).

Effect of selected compounds on human CB CD34$^+$ cell expansion

From our primary screening, we selected 12 compounds that improved HSPC/HSC expansion for further evaluation. Given that ROS act as secondary messengers in intracellular signaling[43–45] and oxidative stress resulting from increased ROS levels may activate stress-induced pathways such as mitogen-activated protein kinases (MAPK; particularly p38 MAPKs, JNKs, and ERKs),[46–48] we hypothesized that MAPK pathways inhibitors might improve HSPC/HSC expansion in 3a medium. Therefore, we tested inhibitors of these pathways: C7 (p-38 inhibitor), U0126 (MEK1/2 kinase inhibitor), and SP600125 (JNK inhibitor) which were implicated in improving HSPC/HSC expansion.[17–19] As lipid peroxidation is the driver of ferroptosis[49–55] and there is an increased lipid peroxidation in 3a medium, we also included compounds (such as ferrostatin-1 [Fer-1] and UAMC-3203 [ferroptosis inhibitor], diethyl maleate [DM; inducer of system XC-transporter], and deferoxamine [DFO; iron chelator]) that can prevent ferroptosis induction in our secondary screening for further study (Figure 4A). Most of these compounds promoted CB CD34$^+$ cell proliferation, except HBED, SP600125, and DFO compared to 3a medium (Figure 4A). Immunophenotype analysis showed that supplementation of Fer-1 significantly increased the percentage of the immunophenotypic HSCs (CD34$^+$CD45RA$^-$CD201$^+$) as compared to vehicle (DMSO)-treated control group (Figure 4B). To assess the intracellular ROS levels and membrane peroxidation status, we co-stained the cells with CellROX Deep Red and BODIPY 581/591 C11, respectively, together with antibodies for HSC markers. Our analyses showed that, as compared to vehicle (DMSO)-treated control group, most of these selected compounds (Figure 4A) resulted in reduced lipid peroxidation, characterized by the reduction in the oxidized BODIPY (BODIPY-ox) positive cells (Figure 4C) and BODIPY-ox staining intensity (Figure S4A). In addition, we observed a decrease in intra-cellular ROS levels (reduction in CellROX positive cells) with some of these compounds (Figures 4D and S4B). Taken together, these data suggest that antioxidant supplementation (in particular those reducing lipid peroxidation) in 3a medium may improve CD34$^+$ HSPC/HSC expansion, likely by lowering the cellular ROS and suppressing the lipid peroxidation. In particular, the ferroptosis inhibitor Fer-1 significantly increased HSC percentage (approximately 2-fold) and moderately improved CD34$^+$ cell expansion compared to DMSO-treated control cells.

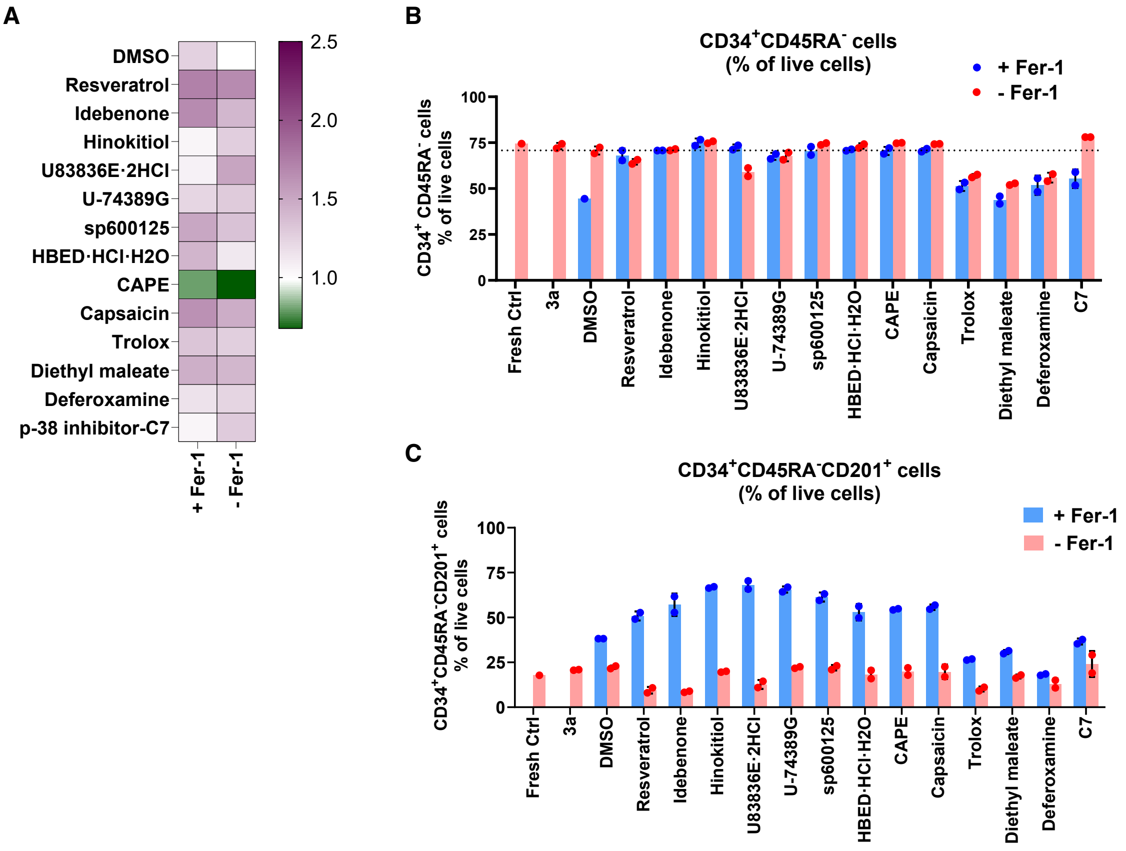

Figure 5. Effect of selected compounds and Fer-1 combination on human CB CD34$^+$ cell expansion

Human CB CD34$^+$ cells were seeded in 96-well plates at 10,000 cells per well in 3a medium with selected compounds with or without Fer-1. (A) Relative cell proliferation (fold change) at day 14 compared to untreated cells. Pooled data from three independent experiments, each performed with unique CB donor cells, are shown. (B and C) HSPC (B) and HSC (C) percentage in live cells with indicated compounds with or without Fer-1. Data represent three independent experiments, each performed with unique CB donor cells.

Effect of selected compounds and Fer-1 combination on human CB CD34$^+$ cell expansion

To explore any additive/synergistic effects among these compounds, we decided to test Fer-1 in combination with these compounds. CB CD34$^+$ cells were treated with selected compounds at optimized concentration with or without Fer-1 in 3a medium for 2 weeks. Most of these compounds except CAPE promoted CD34$^+$ cell expansion, particularly in combination with Fer-1 (Figure 5A). When we compared the HSPC fraction in *ex vivo* expanded cells, we did not observe any major differences for each of the tested compounds with or without Fer-1 supplementation (Figure 5B). Though these compounds individually did not support robust/preferential expansion of immunophenotypic HSCs, to our surprise, most of these compounds except deferoxamine resulted in a preferential expansion of immunophenotypic HSCs when co-treated with Fer-1 during *ex vivo* expansion (Figure 5C). These data indicate that Fer-1 together with other antioxidants is very effective in maintaining immunophenotypic HSC expansion in 3a medium. Though hinokitiol (HK) in combination with Fer-1 (hereafter FHK) did not robustly enhanced cell proliferation (Figure 5A), based on immunophenotypic data, we decided to further interrogate FHK, which consistently retained immunophenotypic HSC during *ex vivo* culture (Figure S5).

Effect of hinokitiol and Fer-1 combination on immunophenotypic HSC expansion

We next investigated the effect of FHK on *ex vivo* expansion of CB CD34$^+$ cells. CB CD34$^+$ cells were cultured in 6-well plates at 300,000 cells/well in 2mL of 3a medium supplemented with 10 μM Fer-1 and 0.5 μM HK for 14 days. DMSO-treated wells were used as vehicle control. We found that FHK-treated CB CD34$^+$ cells showed increased proliferation (~1.5-fold) over DMSO-treated control cells (Figure 6A), indicating the FHK combination promotes CB CD34$^+$ cell expansion in 3a medium. Immunophenotype analysis at day 14 of *ex vivo* culture showed no significant differences in HSPC (CD34$^+$CD45RA$^-$) percentage between DMSO control and FHK-treated cells (Figure 6B). However, we observed immunophenotypic HSCs (CD34$^+$CD45RA$^-$CD201$^+$) percentage was significantly higher in FHK-treated cells as compared to DMSO-treated control cells (Figure 6C). Freshly thawed CB CD34$^+$ cells from respective donors

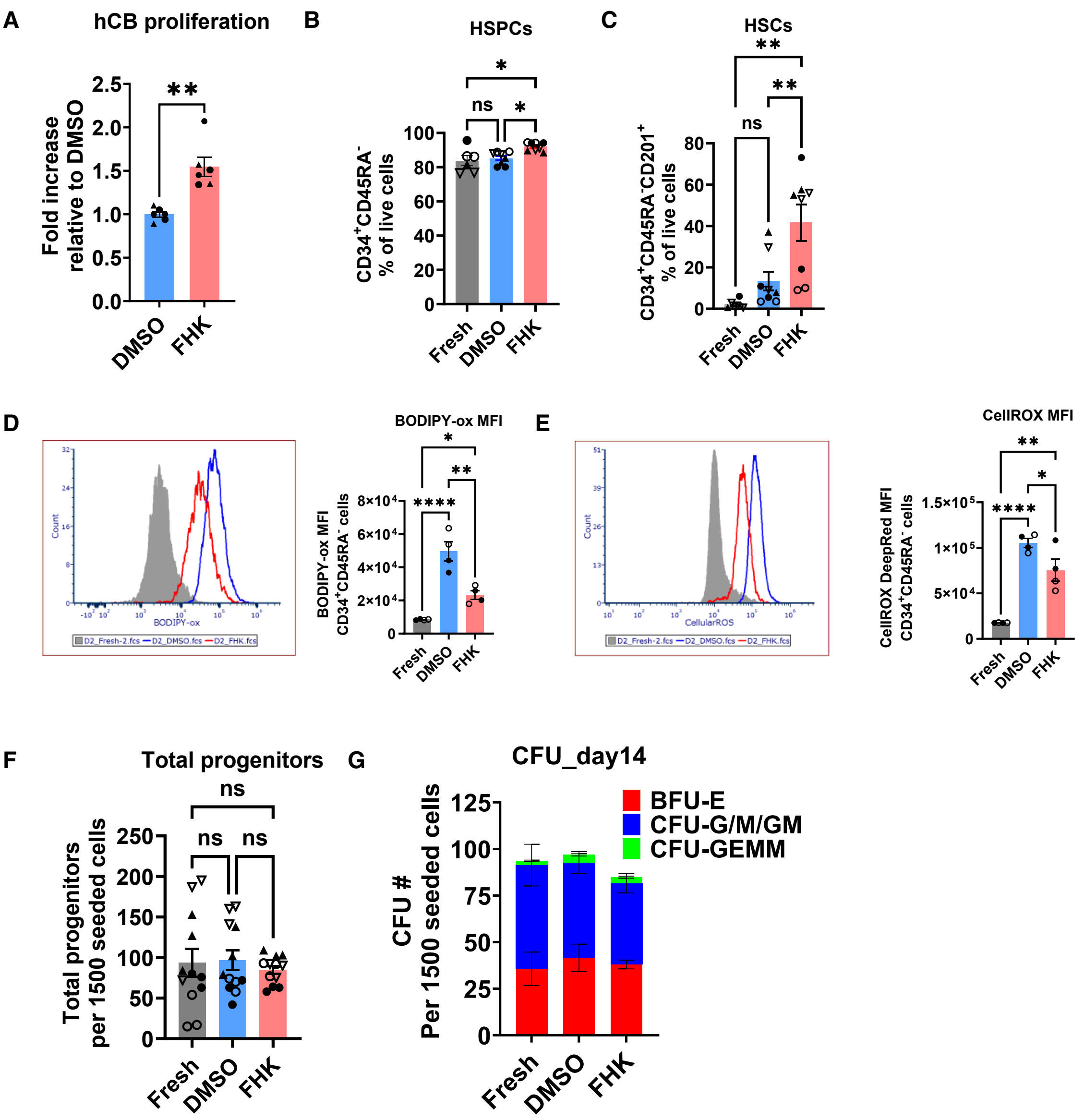

Figure 6. Effect of Fer-1 and hinokitiol combination (FHK) on human CB CD34+ cell expansion

Human CB CD34+ cells were seeded in 6-well plates at 300,000 cells per well in 3a medium with DMSO (vehicle control), or Fer-1 (10 μM) plus hinokitiol (0.5 μM) (FHK). (A) Relative cell proliferation at day 14 compared to DMSO. Pooled data from 3 independent experiments performed in duplicate. Mean ± SEM, ** $p < 0.01$ by *t* test. (B and C) Immunophenotypic analysis showing percentage of human HSPC (B) and HSC (C) in *ex vivo* expanded CB CD34+ cells at day 14. Pooled data from 3 independent experiments with CD34+ cells from 4 unique CB donors, performed in duplicate. (D and E) Lipid peroxidation (D) and intracellular ROS (E) levels in *ex vivo* expanded CD34+CD45RA− cells measured by BODIPY 581/591 C11 and CellROX Deep Red staining, respectively. Representative overlaid histograms (left) and mean fluorescence intensity (MFI, right) are shown. Pooled data from 2 independent experiments with CD34+ cells from 2 unique CB donors, performed in duplicate. (F and G) Colony forming unit (CFU) activity in *ex vivo* expanded human CB CD34+ cells cultured for 14 days. Colony count of total progenitors (F) and various types of colonies (G) were quantified. Pooled data from 3 independent experiments with CD34+ cells from 4 unique CB donors, performed in duplicate. Mean ± SEM. * $p < 0.05$; ** $p < 0.01$; **** $p < 0.0001$ by one-way ANOVA with Tukey's multiple-comparison test except (G). Each CB donor is represented by a unique symbol.

showed very low frequency of CD34+CD45RA-CD201+ cells (Figure 6C). We also looked at CD133 expression on *ex vivo* expanded CB CD34+ cells. CD133, also known as Prominin-1, is a stem cell marker particularly in the hematopoietic and neural lineages. We observed a decrease in the percentage of CD34+CD45RA-CD133+ cells in the *ex vivo* cultured cells compared to freshly thawed CB CD34+ cells (Figure S6A) indicating that FHK supplementation was not effective in maintaining CD133 expression

during *ex vivo* expansion. No significant difference on CD34$^+$ CD45RA$^-$CD201$^+$CD133$^+$ cells was observed among different groups (Figure S6B).

Effect of Fer-1 and hinokitiol combination on lipid peroxidation and intracellular ROS

After establishing that FHK supplementation modestly improves CB CD34$^+$ cells proliferation and preferentially expands CD34$^-$$^+$CD45RA$^-$CD201$^+$ HSCs in 3a medium, we decided to interrogate effect of FHK combination on lipid peroxidation and intracellular ROS levels. *Ex vivo* expanded cells were co-stained with BODIPY and CellROX Deep Red to measure lipid peroxidation and intracellular ROS levels, respectively. We observed the BODIPY staining was significantly higher in *ex vivo* expanded cells in 3a medium compared to freshly thawed CB CD34$^+$ (Figure 6D) indicative of increased lipid peroxidation. Similarly, CellROX Deep Red staining revealed significantly higher levels of intracellular ROS in *ex vivo* expanded cells compared to freshly thawed CB CD34$^+$ (Figure 6E). We found that FHK supplementation in 3a medium resulted in significant reduction in lipid peroxidation (Figure 6D) and intracellular ROS levels (Figure 6E). Although, FHK supplementation did not reduce the lipid peroxidation and intracellular ROS to the level seen with freshly thawed CD34$^+$ cells (Figures 6D and 6E). In addition to measuring lipid peroxidation and intracellular ROS, we also evaluated the intracellular glutathione (GSH) and oxidized glutathione (GSSG) level at day 14 of *ex vivo* expansion. Compared to freshly thawed CB CD34$^+$ cells, we observed a significant increase in GSH to GSSG levels in *ex vivo* expanded cells in 3a medium and FHK supplementation did not alter GSH:GSSG ratio (Figure S7).

Effect of Fer-1 and hinokitiol combination on differentiation potential of CD34$^+$ cells

To evaluate the impact of FHK supplementation on the differentiation potential of *ex vivo* expanded CB CD34$^+$ cells, we performed *in vitro* colony-forming unit (CFU) assay. We first cultured CB CD34$^+$ cells in 3a medium supplemented with FHK for 2 weeks in 6 well plates, using DMSO-treated cells as vehicle control. After 2 weeks of *ex vivo* expansion, expanded cells were plated in Methocult medium without FHK supplementation and were cultured for additional 14 days. At 14 days of differentiation, colonies were counted on STEMVision automated CFU assay reader. We did not observe any significant differences in either total colony counts (Figure 6F) or colony type frequency (BFU-E, CFU-G/M/GM, and CFU-GEMM) (Figures 6G and S8). CFU assay data indicate that FHK supplementation in 3a medium did not compromise or alter the differentiation potential of CB CD34$^+$ cells.

In vivo engraftment and reconstitution potential of FHK-treated CD34$^+$ cells

To evaluate the *in vivo* engraftment and multi-lineage reconstitution potential of *ex vivo* expanded CB CD34$^+$ cells in the presence of FHK, we performed xenotransplantation experiments using NOG-EXL immunodeficient mice. 50,000 fresh or *ex vivo* expanded CB CD34$^+$ cells in 3a medium supplemented either with DMSO (vehicle control) or FHK were injected intravenously into sub-lethally irradiated NOG-EXL immunodeficient mice. Following transplantation, mice were bled at a 4-week interval and peripheral blood was analyzed by flow cytometry to monitor human cell chimerism in transplanted mice (Figure 7A). At week 24 post-transplantation, mice were sacrificed, cells were isolated from bone marrow and spleen, and cells were analyzed for human cell chimerism. In our xenotransplantation experiments, unmanipulated, freshly thawed CB CD34$^+$ cells displayed robust reconstitution with human cells over the 24 weeks post-transplantation observation (Figure 7B). Mice transplanted with *ex vivo* expanded cells showed slower reconstitution kinetics and lower human cell chimerism compared to fresh cells (Figure 7B). Both DMSO-treated control and FHK supplemented group reached comparable levels of human cell chimerism toward the end of the study period (week 20 and beyond). However, there were noticeable differences in the reconstitution kinetics between two groups. Though we observed higher human cell chimerism at the early stage from week 4 to 12 for DMSO-treated control group, mice engrafted with FHK-treated cells achieved higher human cell chimerism exceeding that of DMSO-treated control group at later time points. Mice transplanted with FHK-treated cells showed comparable human cell chimerism in peripheral blood to that of fresh CB cells at the 20th week. Lineage distribution analyses in the peripheral blood of the transplanted mice at week 20 and 24 showed comparable lineage contribution of lymphoid and myeloid cells among all three groups (Figure 7C). Multi-lineage human cell reconstitution was also observed in the spleen (Figure 7D) and the bone marrow (Figure 7E) of transplanted mice at week 24 post-transplantation. Lineage distribution data in peripheral blood, spleen, and bone marrow from individual mouse are shown in Figure S9. Bone marrow analyses at week 24 post-transplantation showed comparable level of HSPCs and HSCs in DMSO- and FHK-treated groups (Figure 7F). Taken together, these data suggest that engraftment and differentiation potential of CB CD34$^+$ HSPC/HSC remained unaffected by FHK treatment.

DISCUSSION

By replacing cytokines and albumin with chemical agonists, Sakurai et al. developed a fully chemically defined cytokine-free medium called 3a medium that supports long-term expansion/maintenance of human CB HSCs.[25] However, following *ex vivo* expansion only 2% of cells retained HSC marker expression that suggests 3a medium is mainly supporting HSPCs expansion. Moreover, HSPCs expanded in 3a medium exhibit increased lipid peroxidation and oxidative stress-induced DNA damage. Given that polyunsaturated fatty acids in lipid membranes are highly sensitive to ROS-mediated oxidation called lipid peroxidation,[52–55] we hypothesized that inclusion of antioxidant(s) may improve HSC expansion in 3a medium. Indeed, we found that trolox supplementation slightly improved cell proliferation and HSC expansion in 3a medium. Since trolox is a hydrophilic vitamin E analog known for its potent antioxidative properties by preventing the generation of lipid peroxides,[56] slightly improved CB CD34$^+$ HSPC proliferation in 3a medium with trolox supplementation is likely attributed to the prevention of lipid peroxidation. We

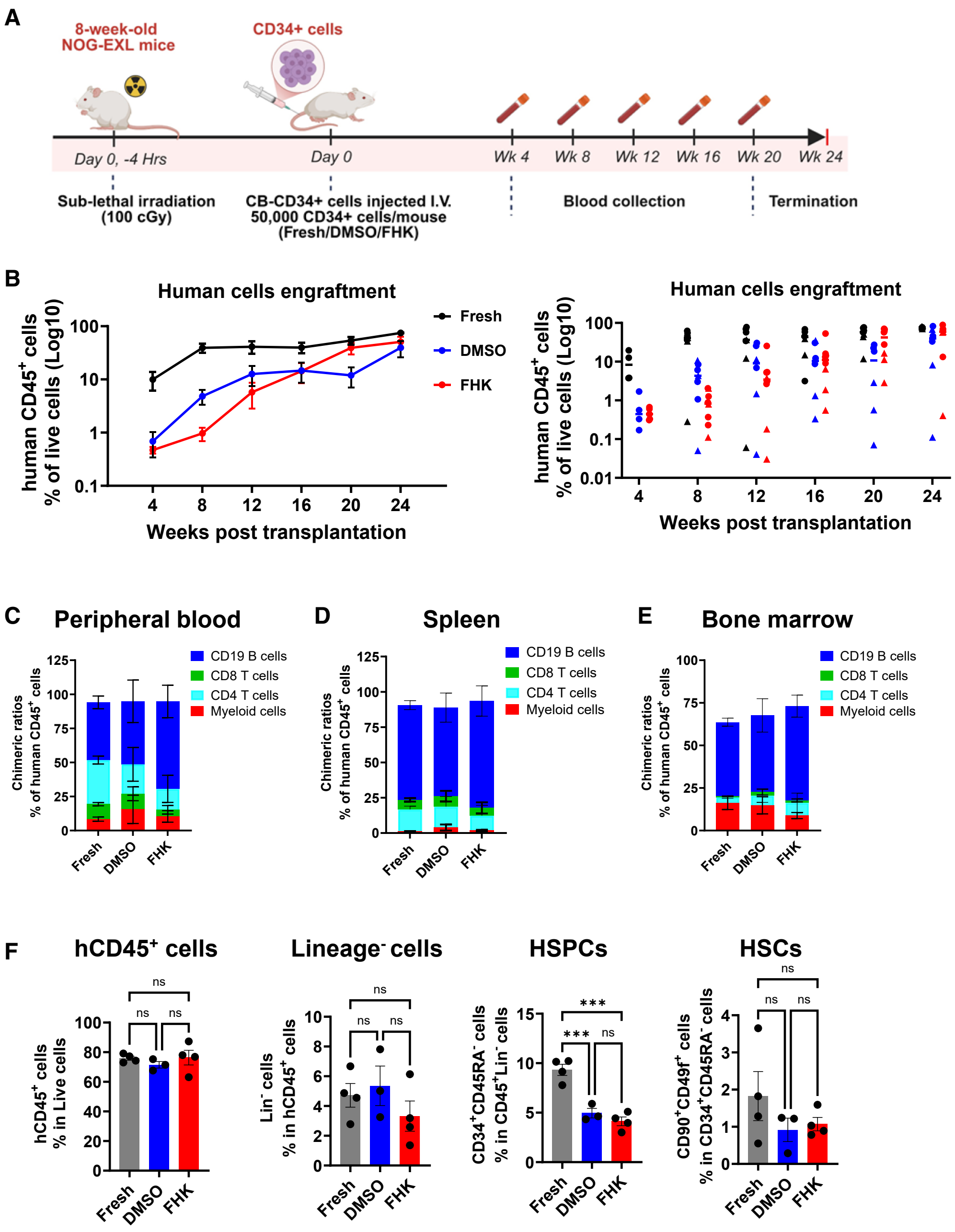

Figure 7. Expanded human CB cells engraftment and chimerism after transplantation

(A) Schematic outlining the xenotransplantation experiment in NOG-EXL mice. Schematic created in BioRender.com. (B) Human blood cell chimerism (CD45$^+$ cell percentage) in peripheral blood at indicated time point (left: pooled data, right: individual mouse data. (C–E) Human blood cell lineage distribution (chimerism ratio) at week 24 in peripheral blood (C), spleen (D), and bone marrow (E) of transplanted mice. Pooled data from two independent experiments performed with expanded CD34$^+$ cells from two unique CB donors (represented by unique symbol ● and▲). Fresh cells from each donor were used as control for comparison. Experiment#1, N = 3 mice per group. Experiment#2, N = 4 (fresh), 4(DMSO), and 5(FHK). Mean ± SEM, two-way ANOVA with Tukey's multiple-comparison test (right). *$p < 0.05$; **$p < 0.01$; ***$p < 0.001$. (F) Bone marrow analyses at week 24 post-transplantation showing human CD45$^+$ cells, lineage$^-$cells, HSPCs, and HSC distribution. Data from one transplantation experiment are shown. Mean ± SEM, one-way ANOVA with Tukey's multiple-comparison test. ***$p < 0.001$.

extended our findings by screening 83 antioxidants/prooxidants and identified 12 antioxidants (including trolox) that improved HSPC/HSC expansion in 3a medium. In fact, resveratrol was the most effective compound in improving *ex vivo* expansion of CB CD34$^+$ cells in 3a medium. Resveratrol is known to improve *ex vivo* expansion of CB CD34$^+$ cells by downregulating intracellular ROS.[14] Interestingly,

in an *in vitro* liposome model system, resveratrol was also shown to prevent the Fe^{2+} catalyzed lipid hydroperoxide-dependent peroxidation more efficiently than trolox.[57] Resveratrol inhibits lipid peroxidation mainly by scavenging lipid peroxyl radicals within the membrane[57–61] and it is likely that improved CB $CD34^+$ cell expansion in 3a medium with resveratrol supplementation is due to suppression of lipid peroxidation. We further show that antioxidant supplementation, particularly those preventing lipid peroxidation (trolox, resveratrol, etc.), resulted in enhanced or preferential expansion of HSC in 3a medium, likely by ameliorating oxidative stress resulting from lipid peroxidation. Our findings further corroborate the supplementation of antioxidants in 3a medium to enhance *ex vivo* expansion of $CD34^+$ HSPCs/HSCs.

Our secondary screening with 19 compounds showed that most of these compounds enhanced HSPC/HSC expansion in 3a medium with more than 2-fold increase in cell yield compared to DMSO-treated control. Interestingly, supplementation of Fer-1 resulted in preferential expansion of immunophenotypic HSCs ($CD34^+CD45RA^-CD201^+$ cells). When tested in combination with Fer-1, most of these compounds resulted in an increase in cell number. Though there was no difference in HSPC ($CD34^+CD45RA^-$) fraction for most of the compounds with or without Fer-1, most of them resulted in an increase in HSC ($CD34^+CD45RA^-CD201^+$ cells) fraction when combined with Fer-1. Notably, preferential expansion of HSC was observed with 9 compounds in combination with Fer-1. None of the 9 compounds maintained the HSC phenotype when tested alone

Further extensive analyses with FHK combination showed consistent and reproducible expansion of HSCs in multiple experiments with CB from independent donors. FHK supplementation in 3a medium resulted in significant increase in cell yield (approximately 1.5-fold over DMSO control). Though the HSPC fraction remained the same, FHK supplementation significantly increased the frequency of HSC in *ex vivo* expanded cells. Moreover, FHK addition resulted in significant reduction in lipid peroxidation and ROS accumulation. Taken together, these data indicate that FHK supplementation significantly improved CB HSC expansion in 3a medium by ameliorating ROS accumulation and suppressing lipid peroxidation. In fact, a recently published study suggests that the antioxidant TEMPOL protects human HSC from culture-induced oxidative stress and prevents loss of functions during *ex vivo* culture.[62] Similar findings were also reported with mouse HSCs in recently published studies[63–66] that indicate deleterious effect of ROS on HSC functions and hematopoietic regeneration. Wang et al. showed that ferumoxytol (FMT)—a catalase-like ROS scavenger—improved expansion of mouse Lin^-, $Sac\text{-}1^+$, $ckit^+$ (LSK cells; 2.6-fold), and phenotypic LT-HSCs (4.5-fold) in long-term culture by reducing intracellular ROS and hydrogen peroxide (H_2O_2) levels and by protecting HSCs from H_2O_2-induced cytotoxicity.[65] In addition, FMT promoted rapid hematopoietic regeneration in mice treated with 5-FU and significantly prolonged their survival. Zhou et al. showed that elevated ROS levels due to abnormal mitochondrial permeability transition pore opening in *Nynrin* knockout mice resulted in diminished HSC frequency, dormancy, and self-renewal.[66] Similarly, loss of mitochondrial connexin 43 (Cx43) caused mitochondrial autophagy, ROS accumulation, and increased AMPK activity that led to HSC senescence/apoptosis and diminished hematopoietic regeneration.[63,64] Our findings and published studies corroborate the beneficial effects of antioxidant supplementation during *ex vivo* expansion of CB HSCs. Preferential expansion of HSCs that we observed with FHK supplementation in a chemically defined 3a-medium has not been reported earlier. Moreover, emerging evidence suggests that iron metabolism play a critical role in HSC biology[67] and HSCs are particularly susceptible to ferroptosis-induced cell death.[68] Among immature hematopoietic cells, HSPCs contain very limited labile iron pool (LIP) and activation of the limited iron response increases regeneration whereas increased cytoplasmic LIP is associated with age-associated decline in HSC functions.[67] Systemic iron overload has a suppressive effect on hematopoiesis, results in markedly decreased HSC functions, impaired engraftment in bone marrow, increased exit from quiescence, and HSC exhaustion[69,70] and is frequently associated with myelodysplastic syndrome (MDS).[71,72] Furthermore, under reduced protein synthesis (such as histone deubiquitinase MYSM1 deficiency), HSCs are highly susceptible to ferroptosis.[68] This susceptibility to ferroptosis can be abrogated by blocking ferroptosis even under low protein synthesis rates.[68] Our findings show that ferroptosis inhibitor Fer-1 alone or in combination with iron chelator hinokitiol resulted in preferential expansion of HSCs in 3a medium. It is plausible that in a fully chemically defined medium (such as 3a medium) devoid of serum/protein supplement, HSCs may have low protein synthesis rate and/or are exposed to oxidative stress resulting from lipid peroxidation that may initiate ferroptosis cascade. Thus, supplementation of FHK in 3a medium supports preferential expansion of HSCs. Our findings are strongly and independently corroborated by a recent published study which demonstrated that ferroptosis inhibition with liproxstatin-1 or ferrostatin-1 markedly enhances human HSC expansion in both serum-free as well as chemically defined culture conditions.[73] Ferroptosis blockade triggers upregulation of ribosome biogenesis and cholesterol synthesis pathways, leading to increased levels of 7-dehydrocholesterol, a potent endogenous ferroptosis inhibitor that itself promotes HSC expansion.

Although lowering oxidative stress is generally expected to enhance HSC functions, we observed no significant difference in the colony-forming stem and progenitor cell frequency in cells expanded under FHK supplementation to that of fresh CB or 3a medium. In addition, colony type (BFU-E, CFU-G/M/GM, and CFU-GEMM) distribution remained unaffected by FHK treatment. These data suggest that FHK supplementation does not adversely affect stem and progenitor cells colony forming and multilineage differentiation potential. It is important to note that *in vitro* CFU assays primarily assess the colony forming and lineage differentiation potential of individual HSPC rather than the long-term repopulating capacity of HSCs. Almost 95% of colony forming activity resides within the $CD34^+$ compartment and colony forming cell frequency exceeds HSC frequency.[74,75] Serke et al. estimated that about 25% of the $CD34^+$ cells

were clonogenic in CFU assay.[76] *In vitro* CFU readouts are confounded by short-term progenitors that preclude the accurate estimation of HSC number/frequency in *in vitro* CFU assay. Therefore, the lack of change in CFU output following FHK treatment likely reflects the unaltered frequency of HSPCs rather than impaired HSC functions. Our *in vivo* xenotransplantation data showed that fresh CB $CD34^+$ HSPCs exhibited robust peripheral chimerism from very early time points whereas *ex vivo* expanded cells (both DMSO control and FHK group) showed slower peripheral blood chimerism. Interestingly, mice transplanted with FHK supplemented cells showed delayed peripheral blood output compared to fresh or DMSO control with gradual increase in human $CD45^+$ cells in peripheral blood reaching to the level of chimerism achieved by fresh or DMSO group. The slower reconstitution kinetics seen with FHK-treated cells could be due to higher percentage of long-term HSCs/HSPCs. In fact, clonal tracking studies of hematopoietic reconstitution in humans after transplantation suggest that hematopoietic reconstitution occurs in two distinct waves with short term HSPCs contributing to early hematopoietic output whereas steady-state hematopoiesis is sustained by long-term HSCs and multipotent progenitors.[77] Studies on non-human primates (NHP) suggest that LT-HSCs actively contribute to early hematopoietic output with little evidence for clonal succession after initial hematopoietic reconstitution.[78,79] Short-term hematopoietic recovery and robust multilineage hematopoiesis following transplantation in NHP is largely driven by $CD34^+CD45RA^-CD90^+$ cells that is highly enriched for HSCs.[80] Though short-lived, cell-type-restricted clones were observed immediately after engraftment, a rapid decrease in clonal diversity with gradual expansion of most of the persisting LT-HSCs and gradual stabilization of hematopoietic output suggests gradual expansion of LT-HSCs following transplantation. Our data indicate gradual expansion of LT-HSC and steady increase in hematopoietic output that stabilized around 20-week post-transplantation. Bone marrow analyses at week 24 post-transplantation showed comparable level of HSPCs and HSCs in mice transplanted with DMSO-treated and FHK-treated cells that indicate FHK treatment does not affect HSPCs/HSCs functions. While we have extensively studied FHK supplementation in this study, it is likely that other compounds together with Fer-1 may have better or comparable hematopoietic engraftment kinetics. Additional studies are required to understand large-scale/extended expansion of HSCs in 3a medium with antioxidant supplementation, hematopoietic reconstitution kinetics, and clonal diversity. FHK could be used to improve the CB $CD34^+$ HSC expansion under chemically defined culture condition and could be used to increase the total nucleated cell number for cord units with low cell number thereby increasing accessibility to HSCT for a significant number of patients.

MATERIALS AND METHODS

Materials and reagents

SCREEN-WELL REDOX library (Catalog # BML-2835) was purchased from Enzo Life Sciences (Farmingdale, NY). CellTiter-Glo Luminescent Cell Viability Assay kit (Catalog #G7571) and GSH/GSSG-Glo assay reagent (Catalog #V6611) were purchased from Promega (Madison, WI). Iscove's modified Dulbecco's medium (IMDM, Catalog # 12440053), insulin-transferrin-selenium-ethanolamine (ITS-X, Catalog #51500056), penicillin-streptomycin (P/S, Catalog # 15140122), human thrombopoietin (hTPO, Catalog # 300-18) and human stem cell factor (hSCF, Catalog # 300-07), lipid peroxidation sensor BODIPY 581/591 C11 (Catalog # D3861), CellROX Deep Red Flow Cytometry Assay reagent (Catalog # C10422), ViaStain AOPI Staining Solution (Catalog # cs2-0106), and HBSS (Catalog # 14025092) were purchased from Thermo Fisher Scientific (Waltham, MA). ferrostatin-1 (Catalog # HY-100579) and Butyzamide (Catalog # HY-148748) were purchased from MedChemExpress (Monmouth Junction, NJ). UM729 (Catalog # 72332), Methocult H4435 enriched (Catalog # 04435), and SmartDish meniscus-free 6-well culture plates (Catalog # 27370) were purchased from STEMCELL Technologies. 740Y-P (Catalog #S7865) was purchased from Selleck Chemicals (Houston, TX). Soluplus (Catalog #E1200) sample was a kind gift from BASF Pharma (Florham Park, New Jersey). EDTA-coated tubes (Micro sample tubes EDTA K3E, 1.3 mL, Screw Cap, Catalog # 41.1395.105) were purchased from Sarstedt Inc. TBMNK Flow Cytometry Assay Kit (Catalog #: 137-00021) was purchased from RayBiotech (Peachtree Corners, GA). Flow cytometry reagents and antibody information are listed in Table S1.

Cell line and animals

Jurkat cell, clone E6-1 was purchased from ATCC (TIB-152). Human CB $CD34^+$ cells were purchased from HemaCare (CB34C-3) and STEMCELL Technologies (200-0001). The NOG-EXL (hGM-CSF/hIL-3 NOG; model # 13395-F) immunodeficient mice were purchased from Taconic Biosciences (Germantown, New York). This study was conducted under an approved institutional review board (IRB) exempt status protocol utilizing deidentified human CB samples purchased from commercial suppliers. Animal studies were conducted under an approved animal study protocol by Institutional Animal Care and Use Committee (IACUC).

Cell culture media

The 3a medium utilized for the expansion of human CB HSCs is a chemically defined medium.[25] 3a medium is composed of basal IMDM, supplemented with 1% ITS-X, 1% P/S, 0.1% Soluplus, 1 μM 740Y-P, 0.1 μM Butyzamide, and 700 nM UM729. Culture medium was changed every 3–4 days with freshly prepared 3a medium. The cytokine medium employed for HSPC expansion was comprised of IMDM, supplemented with 1% ITS-X and 1% P/S, 0.1% Soluplus, 100 ng/mL hTPO, and 10 ng/mL hSCF. Culture medium was changed every 3–4 days with freshly prepared medium.

The CellTiter-Glo luminescent cell viability assay

50 μL of cell suspension per well were plated in opaque-walled 96-well plates. Control wells with medium only were used to measure background luminescence. The plates are equilibrated at room temperature for approximately 30 min. An equal volume (50 μL) of CellTiter-Glo Reagent is then added to each well. After a 10-min incubation at room temperature in the dark, luminescence is recorded

using a luminometer GloMax Explorer Plate Reader (Promega, Madison, WI).

CFU assay

The differentiation potential of the *ex vivo* expanded CB CD34$^+$ cells were assessed using CFU assay. Briefly, human CB cells were cultured with designed treatments for 14 days before CFU assay was initiated. 1,500 cultured cells per well were seeded in Methocult H4435 enriched in a SmartDish meniscus-free 6-well culture plates and cultured at 37°C with 5% CO_2 for another 14 days. Plates were read at day 14 with STEMvision Automated CFU Assay Reader and analysis system (STEMCELL Technologies) following the manufacturer instructions.

Lipid peroxidation and intracellular level ROS analysis

Lipid peroxidation and intracellular ROS levels were assessed using BODIPY 581/591 C11 and CellROX Deep Red Flow Cytometry Assay reagent, respectively. Briefly, cells were stained directly in culture media with 1 μM BODIPY and 5 μM CellROX for 1 h at 37°C. For positive controls, cells were treated with 100 μM tert-butyl hydroperoxide (tBHP) for 1 h prior to staining to induce ROS production. Cells were treated with 10 μM trolox for 1 h to assess reduction in BODIPY oxidation. Cells were then washed twice with PBS and incubated with Zombie Yellow viability dye for 15 min, according to the manufacturer's instructions. Subsequently, cells were incubated with a cocktail of antibodies diluted in cell staining buffer at 4°C for 45 min. Following two washes with staining buffer, cells were analyzed on a Cytek Northern Lights spectral flow cytometer using SpectroFlo software (Cytek Biosciences). Data were analyzed using FCS Express software (De Novo Software).

Xenotransplantation in NOG-EXL mice

The engraftment potential of *ex vivo* expanded CB CD34$^+$ cells were evaluated by performing xenotransplantation in NOG-EXL mice. CD34$^+$ cells were either freshly thawed or cultured for 14 days in the presence of vehicle (DMSO) or a combination of ferrostatin-1 (Fer-1) and hinokitiol (HK) in 6-well plates at a density of 3×10^5 cells/mL with appropriate media changes every 3–4 days. Following expansion, cells were harvested, pooled, and counted. A subset of 1×10^5 cells was stained for immunophenotyping using antibodies against human CD34, CD201, CD45RA, CD133, and lineage markers. Prior to transplantation, NOG-EXL recipient mice were exposed to a sub-lethal dose of 100 cGy ɣ-irradiation using a Cesium-137 radiation source (J.L. Shepherd Mark I) to facilitate engraftment. Cultured cells from each condition were washed and resuspended in HBSS. 4 h post-irradiation, irradiated mice were transplanted with 5×10^4 cells/mouse by tail vein injection. Peripheral blood was collected at 4-, 8-, 12-, 16-, 20-, and 24-weeks post-transplantation and analyzed by flow cytometry to assess human hematopoietic chimerism. The study was terminated at 24 weeks, mice were sacrificed by CO_2 euthanasia, and blood, bone marrow, and spleen were harvested for analyses.

Peripheral blood, bone marrow, and spleen analyses

Peripheral blood from the transplanted NOG-EXL mice was collected at a 4-week interval by submandibular venipuncture (100–200 μL) in EDTA-coated tubes and was analyzed to assess human blood cell reconstitution. Phenotypic analysis was performed using the RayBio Human TBMNK Flow Cytometry Kit, which enables the identification and quantification of mature human lymphocyte and monocyte subsets in whole blood. Anti-mouse CD45 antibody was added to the TBMNK panel to identify mouse blood cells. Red blood cells were first lysed using the red blood cell lysis buffer included in the kit. Following lysis, cells were stained according to the manufacturer's protocol. Flow cytometry was performed to determine the percentage of each human blood cell population in peripheral blood. Following the same process, spleen and bone marrow cells collected at week 24 were analyzed. In addition, at week 24 bone marrow cells were also analyzed to determine the percentage of human HSPC/HSC.

DATA AND CODE AVAILABILITY

Dataset related to this study is presented in the article. Study related raw data are available on Mendeley Data: http://www.doi.org/10.17632/fnmx8wsnwb.1. Material request can be sent to the corresponding author.

ACKNOWLEDGMENTS

We thank Drs. Zhaohui Ye, Brenton McCright, and Steven Oh of the Center for Biologics Evaluation and Research (CBER), FDA, Silver Spring, MD, for critical review of the manuscript and providing helpful comments. We thank Dr. John Dennis, Prosper Tah, and the Division of Veterinary Services (DVS) FDA's laboratory animal care and use program on the White Oak campus for their technical support with NOG-EXL mice care and animal experiments. We thank Dr. Byung Woo Kim for his technical support with the CFU assay. This work was supported by the Advanced Manufacturing Funds and Intramural Research Program of the CBER, FDA. Our comments/contributions are an informal communication and represent our own best judgment. These comments do not bind or obligate FDA. The graphical abstract is created in BioRender.com.

AUTHOR CONTRIBUTIONS

Conceptualization, methodology, and experimental design were performed by P.K.M. Experiments were performed by L.L. and P.K.M. Data analyses were performed by L.L. and P.M. L.L. and P.M. wrote, reviewed, and edited the manuscript.

DECLARATION OF INTERESTS

The authors declare no conflict of interests.

DECLARATION OF GENERATIVE AI AND AI-ASSISTED TECHNOLOGIES IN THE WRITING PROCESS

During the preparation of this manuscript, the authors used FDA's generative AI tool Elsa for writing abstract and for proofreading to identify and correct any errors. After using this tool, the authors reviewed and edited the content as needed and takes full responsibility for the content of the publication.

SUPPLEMENTAL INFORMATION

Supplemental information can be found online at https://doi.org/10.1016/j.omta.2026.201711.

REFERENCES

1. Dumont-Lagacé, M., Feghaly, A., Meunier, M.-C., Finney, M., Van't Hof, W., Frenet, E.M., Sauvageau, G., and Cohen, S. (2022). UM171 expansion of cord blood improves donor availability and HLA matching for all patients, including minorities. Transplant. Cell. Ther. *28*, 410.e411–410.e415.

2. Stiff, P.J., Montesinos, P., Peled, T., Landau, E., Goudsmid, N.R., Mandel, J., Hasson, N., Olesinski, E., Glukhman, E., Snyder, D.A., et al. (2018). Cohort-controlled comparison of umbilical cord blood transplantation using carlecortemcel-L, a single progenitor–enriched cord blood, to double cord blood unit transplantation. Biol. Blood Marrow Transplant. *24*, 1463–1470.

3. De Lima, M., McMannis, J., Gee, A., Komanduri, K., Couriel, D., Andersson, B.S., Hosing, C., Khouri, I., Jones, R., Champlin, R., et al. (2008). Transplantation of ex vivo expanded cord blood cells using the copper chelator tetraethylenepentamine: a phase I/II clinical trial. Bone Marrow Transplant. *41*, 771–778.

4. Peled, T., Shoham, H., Aschengrau, D., Yackoubov, D., Frei, G., Rosenheimer G, N., Lerrer, B., Cohen, H.Y., Nagler, A., Fibach, E., and Peled, A. (2012). Nicotinamide, a SIRT1 inhibitor, inhibits differentiation and facilitates expansion of hematopoietic progenitor cells with enhanced bone marrow homing and engraftment. Exp. Hematol. *40*, 342–355.e1.

5. Horwitz, M.E., Stiff, P.J., Cutler, C., Brunstein, C., Hanna, R., Maziarz, R.T., Rezvani, A.R., Karris, N.A., McGuirk, J., Valcarcel, D., et al. (2021). Omidubicel vs standard myeloablative umbilical cord blood transplantation: results of a phase 3 randomized study. Blood *138*, 1429–1440.

6. Horwitz, M.E., Chao, N.J., Rizzieri, D.A., Long, G.D., Sullivan, K.M., Gasparetto, C., Chute, J.P., Morris, A., McDonald, C., Waters-Pick, B., et al. (2014). Umbilical cord blood expansion with nicotinamide provides long-term multilineage engraftment. J. Clin. Investig. *124*, 3121–3128.

7. Ohishi, K., Varnum-Finney, B., and Bernstein, I.D. (2002). Delta-1 enhances marrow and thymus repopulating ability of human CD34+ CD38–cord blood cells. J. Clin. Investig. *110*, 1165–1174.

8. Delaney, C., Heimfeld, S., Brashem-Stein, C., Voorhies, H., Manger, R.L., and Bernstein, I.D. (2010). Notch-mediated expansion of human cord blood progenitor cells capable of rapid myeloid reconstitution. Nat. Med. *16*, 232–236.

9. Wagner, J.E., Brunstein, C.G., Boitano, A.E., DeFor, T.E., McKenna, D., Sumstad, D., Blazar, B.R., Tolar, J., Le, C., Jones, J., et al. (2016). Phase I/II trial of StemRegenin-1 expanded umbilical cord blood hematopoietic stem cells supports testing as a stand-alone graft. Cell Stem Cell *18*, 144–155.

10. Boitano, A.E., Wang, J., Romeo, R., Bouchez, L.C., Parker, A.E., Sutton, S.E., Walker, J.R., Flaveny, C.A., Perdew, G.H., Denison, M.S., et al. (2010). Aryl hydrocarbon receptor antagonists promote the expansion of human hematopoietic stem cells. Science *329*, 1345–1348.

11. Fares, I., Chagraoui, J., Gareau, Y., Gingras, S., Ruel, R., Mayotte, N., Csaszar, E., Knapp, D.J.H.F., Miller, P., Ngom, M., et al. (2014). Pyrimidoindole derivatives are agonists of human hematopoietic stem cell self-renewal. Science *345*, 1509–1512.

12. Cohen, S., Bambace, N., Ahmad, I., Roy, J., Tang, X., Zhang, M.-J., Burns, L., Barabé, F., Bernard, L., Delisle, J.-S., et al. (2023). Improved outcomes of UM171–expanded cord blood transplantation compared with other graft sources: real-world evidence. Blood Adv. *7*, 5717–5726.

13. Cohen, S., Roy, J., Lachance, S., Delisle, J.-S., Marinier, A., Busque, L., Roy, D.-C., Barabé, F., Ahmad, I., Bambace, N., et al. (2020). Hematopoietic stem cell transplantation using single UM171-expanded cord blood: a single-arm, phase 1–2 safety and feasibility study. Lancet Haematol. *7*, e134–e145.

14. Tang, C., Zhang, W., Cai, H., Ye, Z., Zhang, X., and Tan, W. (2019). Resveratrol improves ex vivo expansion of CB-CD34+ cells via downregulating intracellular reactive oxygen species level. J. Cell. Biochem. *120*, 7778–7787.

15. North, T.E., Goessling, W., Walkley, C.R., Lengerke, C., Kopani, K.R., Lord, A.M., Weber, G.J., Bowman, T.V., Jang, I.-H., Grosser, T., et al. (2007). Prostaglandin E2 regulates vertebrate haematopoietic stem cell homeostasis. Nature *447*, 1007–1011.

16. Cutler, C., Multani, P., Robbins, D., Kim, H.T., Le, T., Hoggatt, J., Pelus, L.M., Desponts, C., Chen, Y.-B., Rezner, B., et al. (2013). Prostaglandin-modulated umbilical cord blood hematopoietic stem cell transplantation. Blood *122*, 3074–3081.

17. Bari, S., Zhong, Q., Fan, X., Poon, Z., Lim, A.S.T., Lim, T.H., Dighe, N., Li, S., Chiu, G.N.C., Chai, C.L.L., and Hwang, W.Y.K. (2018). Ex vivo expansion of CD34+ CD90+ CD49f+ hematopoietic stem and progenitor cells from non-enriched umbilical cord blood with azole compounds. Stem Cells Transl. Med. *7*, 376–393.

18. Xiao, X., Lai, W., Xie, H., Liu, Y., Guo, W., Liu, Y., Li, Y., Li, Y., Zhang, J., Chen, W., et al. (2019). Targeting JNK pathway promotes human hematopoietic stem cell expansion. Cell Discov. *5*, 2.

19. Xie, H., Sun, Z., Xiao, X., Liu, D., Qi, H., Tian, G., Chen, M., Chen, L., and Su, X. (2022). Transient inhibition of the JNK pathway promotes human hematopoietic stem cell quiescence and engraftment. Stem Cells Transl. Med. *11*, 597–603.

20. Li, J., Wang, X., Ding, J., Zhu, Y., Min, W., Kuang, W., Yuan, K., Sun, C., and Yang, P. (2022). Development and clinical advancement of small molecules for ex vivo expansion of hematopoietic stem cell. Acta Pharm. Sin. B *12*, 2808–2831.

21. Ghafouri-Fard, S., Niazi, V., Taheri, M., and Basiri, A. (2021). Effect of small molecule on ex vivo expansion of cord blood hematopoietic stem cells: a concise review. Front. Cell Dev. Biol. *9*, 649115.

22. Baldo, B.A. (2014). Side effects of cytokines approved for therapy. Drug Saf. *37*, 921–943.

23. Liu, C., Chu, D., Kalantar-Zadeh, K., George, J., Young, H.A., and Liu, G. (2021). Cytokines: from clinical significance to quantification. Adv. Sci. *8*, 2004433.

24. Tarrant, J.M. (2010). Blood cytokines as biomarkers of in vivo toxicity in preclinical safety assessment: considerations for their use. Toxicol. Sci. *117*, 4–16.

25. Sakurai, M., Ishitsuka, K., Ito, R., Wilkinson, A.C., Kimura, T., Mizutani, E., Nishikii, H., Sudo, K., Becker, H.J., Takemoto, H., et al. (2023). Chemically defined cytokine-free expansion of human haematopoietic stem cells. Nature *615*, 127–133.

26. Tai-Nagara, I., Matsuoka, S., Ariga, H., and Suda, T. (2014). Mortalin and DJ-1 coordinately regulate hematopoietic stem cell function through the control of oxidative stress. Blood *123*, 41–50.

27. Ludin, A., Gur-Cohen, S., Golan, K., Kaufmann, K.B., Itkin, T., Medaglia, C., Lu, X.-J., Ledergor, G., Kollet, O., Lapidot, T., et al. (2014). Reactive oxygen species regulate hematopoietic stem cell self-renewal, migration and development, as well as their bone marrow microenvironment. Antioxid. Redox Signal. *21*, 1605–1619.

28. Du, W., Adam, Z., Rani, R., Zhang, X., and Pang, Q. (2008). Oxidative stress in Fanconi anemia hematopoiesis and disease progression. Antioxid. Redox Signal. *10*, 1909–1921.

29. Henry, E., Souissi-Sahraoui, I., Deynoux, M., Lefèvre, A., Barroca, V., Campalans, A., Ménard, V., Calvo, J., Pflumio, F., Arcangeli, M.-L., et al. (2020). Human hematopoietic stem/progenitor cells display reactive oxygen species-dependent long-term hematopoietic defects after exposure to low doses of ionizing radiations. Haematologica *105*, 2044–2055.

30. Ito, K., Hirao, A., Arai, F., Takubo, K., Matsuoka, S., Miyamoto, K., Ohmura, M., Naka, K., Hosokawa, K., Ikeda, Y., and Suda, T. (2006). Reactive oxygen species act through p38 MAPK to limit the lifespan of hematopoietic stem cells. Nat. Med. *12*, 446–451.

31. Morales-Hernández, A., Martinat, A., Chabot, A., Kang, G., and McKinney-Freeman, S. (2018). Elevated oxidative stress impairs hematopoietic progenitor function in C57BL/6 substrains. Stem Cell Rep. *11*, 334–347.

32. Owusu-Ansah, E., and Banerjee, U. (2009). Reactive oxygen species prime Drosophila haematopoietic progenitors for differentiation. Nature *461*, 537–541.

33. Cao, Y., Fang, Y., Cai, J., Li, X., Xu, F., Yuan, N., Zhang, S., and Wang, J. (2016). ROS functions as an upstream trigger for autophagy to drive hematopoietic stem cell differentiation. Hematology *21*, 613–618.

34. Jing, Q., Zhou, C., Zhang, J., Zhang, P., Wu, Y., Zhou, J., Tong, X., Li, Y., Du, J., and Wang, Y. (2024). Role of reactive oxygen species in myelodysplastic syndromes. Cell. Mol. Biol. Lett. *29*, 53.

35. Prieto-Bermejo, R., Romo-González, M., Pérez-Fernández, A., Ijurko, C., and Hernández-Hernández, Á. (2018). Reactive oxygen species in haematopoiesis: leukaemic cells take a walk on the wild side. J. Exp. Clin. Cancer Res. *37*, 1–18.

36. Pavitra, E., Acharya, R.K., Gupta, V.K., Verma, H.K., Kang, H., Lee, J.-H., Sahu, T., Bhaskar, L., Raju, G.S.R., and Huh, Y.S. (2024). Impacts of oxidative stress and antioxidants on the development, pathogenesis, and therapy of sickle cell disease: A comprehensive review. Biomed. Pharmacother. *176*, 116849.

37. Atiq, A., Lee, H.J., Khan, A., Kang, M.H., Rehman, I.U., Ahmad, R., Tahir, M., Ali, J., Choe, K., Park, J.S., and Kim, M.O. (2023). Vitamin E analog trolox attenuates MPTP-induced Parkinson's disease in mice, mitigating oxidative stress, neuroinflammation, and motor impairment. Int. J. Mol. Sci. *24*, 9942.

38. Forrest, V.J., Kang, Y.-H., McClain, D.E., Robinson, D.H., and Ramakrishnan, N. (1994). Oxidative stress-induced apoptosis prevented by Trolox. Free Radic. Biol. Med. *16*, 675–684.

39. Guo, C., He, Z., Wen, L., Zhu, L., Lu, Y., Deng, S., Yang, Y., Wei, Q., and Yuan, H. (2012). Cytoprotective effect of trolox against oxidative damage and apoptosis in the NRK-52e cells induced by melamine. Cell Biol. Int. *36*, 183–188.

40. Messier, E.M., Bahmed, K., Tuder, R.M., Chu, H.W., Bowler, R.P., and Kosmider, B. (2013). Trolox contributes to Nrf2-mediated protection of human and murine primary alveolar type II cells from injury by cigarette smoke. Cell Death Dis. *4*, e573.

41. Tasci, T., Orta-Yilmaz, B., Aydin, Y., and Caliskan, M. (2024). N-acetylcysteine attenuates sodium arsenite-induced oxidative stress and apoptosis in embryonic fibroblast cells. Toxicol. Res. *13*, tfae128.

42. Zhang, X., Xu, L., Ma, W., Shi, B., Liu, Q., Song, Y., Fang, C., Liu, P., Qiao, S., Cai, J., and Zhang, Z. (2023). N-acetyl-L-cysteine alleviated the oxidative stress-induced inflammation and necroptosis caused by excessive NiCl2 in primary spleen lymphocytes. Front. Immunol. *14*, 1146645.

43. Forman, H.J., Maiorino, M., and Ursini, F. (2010). Signaling functions of reactive oxygen species. Biochemistry *49*, 835–842.

44. Sinenko, S.A., Starkova, T.Y., Kuzmin, A.A., and Tomilin, A.N. (2021). Physiological signaling functions of reactive oxygen species in stem cells: from flies to man. Front. Cell Dev. Biol. *9*, 714370.

45. Morris, G., Gevezova, M., Sarafian, V., and Maes, M. (2022). Redox regulation of the immune response. Cell. Mol. Immunol. *19*, 1079–1101.

46. Son, Y., Cheong, Y.-K., Kim, N.-H., Chung, H.-T., Kang, D.G., and Pae, H.O. (2011). Mitogen-activated protein kinases and reactive oxygen species: how can ROS activate MAPK pathways? J. Signal Transduct. *2011*, 792639.

47. Dolado, I., Swat, A., Ajenjo, N., De Vita, G., Cuadrado, A., and Nebreda, A.R. (2007). p38α MAP kinase as a sensor of reactive oxygen species in tumorigenesis. Cancer Cell *11*, 191–205.

48. McCubrey, J.A., Lahair, M.M., and Franklin, R.A. (2006). Reactive oxygen species-induced activation of the MAP kinase signaling pathways. Antioxid. Redox Signal. *8*, 1775–1789.

49. Bayır, H., Anthonymuthu, T.S., Tyurina, Y.Y., Patel, S.J., Amoscato, A.A., Lamade, A.M., Yang, Q., Vladimirov, G.K., Philpott, C.C., and Kagan, V.E. (2020). Achieving life through death: redox biology of lipid peroxidation in ferroptosis. Cell Chem. Biol. *27*, 387–408.

50. Yang, W.S., and Stockwell, B.R. (2016). Ferroptosis: death by lipid peroxidation. Trends Cell Biol. *26*, 165–176.

51. von Krusenstiern, A.N., Robson, R.N., Qian, N., Qiu, B., Hu, F., Reznik, E., Smith, N., Zandkarimi, F., Estes, V.M., Dupont, M., et al. (2023). Identification of essential sites of lipid peroxidation in ferroptosis. Nat. Chem. Biol. *19*, 719–730.

52. Endale, H.T., Tesfaye, W., and Mengstie, T.A. (2023). ROS induced lipid peroxidation and their role in ferroptosis. Front. Cell Dev. Biol. *11*, 1226044.

53. Chen, X., Kang, R., and Tang, D. (2021). Ferroptosis by lipid peroxidation: the tip of the iceberg? Front. Cell Dev. Biol. *9*, 646890.

54. Su, L.-J., Zhang, J.-H., Gomez, H., Murugan, R., Hong, X., Xu, D., Jiang, F., and Peng, Z.Y. (2019). Reactive oxygen species-induced lipid peroxidation in apoptosis, autophagy, and ferroptosis. Oxid. Med. Cell. Longev. *2019*, 5080843.

55. Mortensen, M.S., Ruiz, J., and Watts, J.L. (2023). Polyunsaturated fatty acids drive lipid peroxidation during ferroptosis. Cells *12*, 804.

56. Lúcio, M., Nunes, C., Gaspar, D., Ferreira, H., Lima, J.L.F.C., and Reis, S. (2009). Antioxidant activity of vitamin E and Trolox: understanding of the factors that govern lipid peroxidation studies in vitro. Food Biophys. *4*, 312–320.

57. Tadolini, B., Juliano, C., Piu, L., Franconi, F., and Cabrini, L. (2000). Resveratrol inhibition of lipid peroxidation. Free Radic. Res. *33*, 105–114.

58. Lin, M.-C., Liu, C.-C., Lin, Y.C., and Liao, C.S. (2021). Resveratrol protects against cerebral ischemic injury via restraining lipid peroxidation, transition elements, and toxic metal levels, but enhancing anti-oxidant activity. Antioxidants *10*, 1515.

59. Franco, J.G., de Moura, E.G., Koury, J.C., Trotta, P.A., Cordeiro, A., Souza, L.L., Almeida, N.A.d.S., Lima, N.d.S., Pazos-Moura, C.C., Lisboa, P.C., and Passos, M.C.F. (2010). Resveratrol reduces lipid peroxidation and increases sirtuin 1 expression in adult animals programmed by neonatal protein restriction. J. Endocrinol. *207*, 319–328.

60. Xiao, N.-N. (2015). Effects of resveratrol supplementation on oxidative damage and lipid peroxidation induced by strenuous exercise in rats. Biomol. Ther. *23*, 374–378.

61. Zemheri-Navruz, F., Ince, S., Arslan-Acaroz, D., Acaroz, U., Demirel, H.H., and Demirkapi, E.N. (2023). Resveratrol alleviates pyraclostrobin-induced lipid peroxidation, oxidative stress, and DNA damage in rats. Environ. Sci. Pollut. Res. Int. *30*, 6414–6423.

62. Henry, E., Picou, F., Barroca, V., Dechamps, N., Sobrino, S., Six, E., Gobeaux, C., Auberger, P., Hérault, O., Pflumio, F., and Arcangeli, M.L. (2023). The antioxidant TEMPOL protects human hematopoietic stem cells from culture-mediated loss of functions. Stem Cells Transl. Med. *12*, 676–688.

63. Singh, A.K., D'Alessandro, A., Wellendorf, A.M., Gonzalez-Nieto, D., Kofron, M., Dziecíatkowska, M., Mejia, L., Barrio, L.C., Filippi, M.D., and Cancelas, J.A. (2025). Metabolic adaptation of regenerative hematopoiesis depends on docking-independent mitochondrial connexin 43. Blood *146*, 2306–2321. https://doi.org/10.1182/blood.2024028079.

64. Taniguchi Ishikawa, E., Gonzalez-Nieto, D., Ghiaur, G., Dunn, S.K., Ficker, A.M., Murali, B., Madhu, M., Gutstein, D.E., Fishman, G.I., Barrio, L.C., and Cancelas, J.A. (2012). Connexin-43 prevents hematopoietic stem cell senescence through transfer of reactive oxygen species to bone marrow stromal cells. Proc. Natl. Acad. Sci. USA *109*, 9071–9076. https://doi.org/10.1073/pnas.1120358109.

65. Wang, Q., Qian, W., Han, Y., Mao, Y., Gao, Z., Chen, Y., Zeng, X., Lu, H., Jiang, L., Li, J., et al. (2025). Ferumoxytol promotes haematopoietic stem cell post-injury regeneration as a reactive oxygen species scavenger. Nat. Nanotechnol. *20*, 959–969. https://doi.org/10.1038/s41565-025-01907-2.

66. Zhou, C., Kuang, M., Tao, Y., Wang, J., Luo, Y., Fu, Y., Chen, Z., Liu, Y., Li, Z., Wu, W., et al. (2024). Nynrin preserves hematopoietic stem cell function by inhibiting the mitochondrial permeability transition pore opening. Cell Stem Cell *31*, 1359–1375.e8. https://doi.org/10.1016/j.stem.2024.06.007.

67. Kao, Y.-R., Chen, J., Kumari, R., Ng, A., Zintiridou, A., Tatiparthy, M., Ma, Y., Aivalioti, M.M., Moulik, D., Sundaravel, S., et al. (2024). An iron rheostat controls hematopoietic stem cell fate. Cell Stem Cell *31*, 378–397.e12.

68. Zhao, J., Jia, Y., Mahmut, D., Deik, A.A., Jeanfavre, S., Clish, C.B., and Sankaran, V.G. (2023). Human hematopoietic stem cell vulnerability to ferroptosis. Cell *186*, 732–747.e16.

69. Muto, Y., Nishiyama, M., Nita, A., Moroishi, T., and Nakayama, K.I. (2017). Essential role of FBXL5-mediated cellular iron homeostasis in maintenance of hematopoietic stem cells. Nat. Commun. *8*, 16114.

70. Chai, X., Li, D., Cao, X., Zhang, Y., Mu, J., Lu, W., Xiao, X., Li, C., Meng, J., Chen, J., et al. (2015). ROS-mediated iron overload injures the hematopoiesis of bone marrow by damaging hematopoietic stem/progenitor cells in mice. Sci. Rep. *5*, 10181.

71. Jin, X., He, X., Cao, X., Xu, P., Xing, Y., Sui, S., Wang, L., Meng, J., Lu, W., Cui, R., et al. (2018). Iron overload impairs normal hematopoietic stem and progenitor cells through reactive oxygen species and shortens survival in myelodysplastic syndrome mice. Haematologica *103*, 1627–1634. https://doi.org/10.3324/haematol.2018.193128.

72. Lyle, L., and Hirose, A. (2018). Iron Overload in Myelodysplastic Syndromes: Pathophysiology, Consequences, Diagnosis, and Treatment. J. Adv. Pract. Oncol. *9*, 392–405.

73. Della Volpe, L., Lee, A.J., Antoszewski, M., Deik, A.A., Safina, K.R., Gao, T., Guo, C.J., Ye, T., Lyu, P., Martin-Rufino, J.D., et al. (2025). Inhibiting ferroptosis enhances ex vivo expansion of human haematopoietic stem cells. Nat. Cell Biol. *27*, 2214–2224. https://doi.org/10.1038/s41556-025-01814-7.

74. Manz, M.G., Miyamoto, T., Akashi, K., and Weissman, I.L. (2002). Prospective isolation of human clonogenic common myeloid progenitors. Proc. Natl. Acad. Sci. USA *99*, 11872–11877. https://doi.org/10.1073/pnas.172384399.

75. Thompson, E.N., Carlino, M.J., Scanlon, V.M., Grimes, H.L., and Krause, D.S. (2023). Assay optimization for the objective quantification of human multilineage colony-forming units. Exp. Hematol. *124*, 36–44.e3. https://doi.org/10.1016/j.exphem.2023.05.007.

76. Serke, S., Säuberlich, S., Abe, Y., and Huhn, D. (1991). Analysis of CD34-positive hemopoietic progenitor cells from normal human adult peripheral blood: flow-cytometrical

studies and in-vitro colony (CFU-GM, BFU-E) assays. Ann. Hematol. *62*, 45–53. https://doi.org/10.1007/BF01714899.

77. Biasco, L., Pellin, D., Scala, S., Dionisio, F., Basso-Ricci, L., Leonardelli, L., Scaramuzza, S., Baricordi, C., Ferrua, F., Cicalese, M.P., et al. (2016). In vivo tracking of human hematopoiesis reveals patterns of clonal dynamics during early and steady-state reconstitution phases. Cell Stem Cell *19*, 107–119.

78. Radtke, S., Enstrom, M., Pande, D., Duke, E.R., Cardozo-Ojeda, E.F., Madhu, R., Owen, S., Kanestrom, G., Cui, M., Perez, A.M., et al. (2023). Stochastic fate decisions of HSCs after transplantation: early contribution, symmetric expansion, and pool formation. Blood *142*, 33–43.

79. Koelle, S.J., Espinoza, D.A., Wu, C., Xu, J., Lu, R., Li, B., Donahue, R.E., and Dunbar, C.E. (2017). Quantitative stability of hematopoietic stem and progenitor cell clonal output in rhesus macaques receiving transplants. Blood *129*, 1448–1457.

80. Radtke, S., Adair, J.E., Giese, M.A., Chan, Y.-Y., Norgaard, Z.K., Enstrom, M., Haworth, K.G., Schefter, L.E., and Kiem, H.-P. (2017). A distinct hematopoietic stem cell population for rapid multilineage engraftment in nonhuman primates. Sci. Transl. Med. *9*, eaan1145.

OMTA, Volume *34*

Supplemental information

Ferrostatin-1 and hinokitiol supplementation enhance human hematopoietic stem cell expansion in a chemically defined medium

Lushen Li and Pankaj K. Mandal

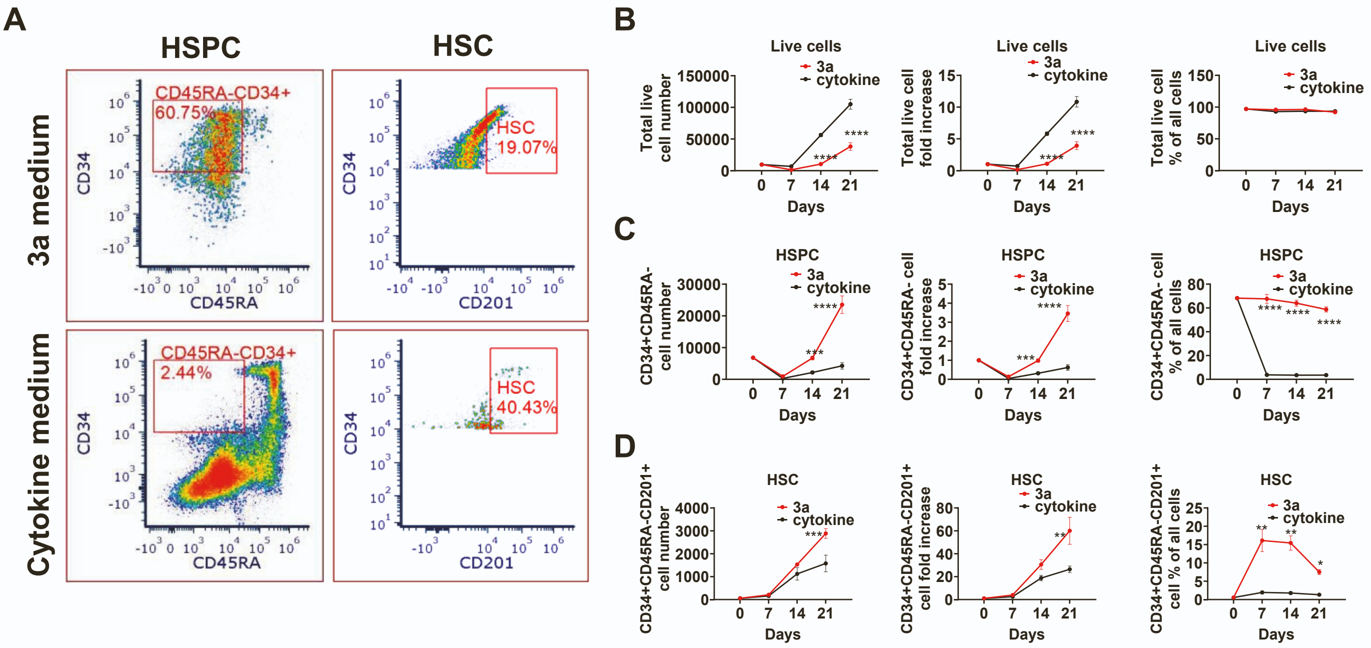

Figure S1: Human cord blood hematopoietic stem cell expansion in 3a medium. Human cord blood CD34+ cells were seeded in 96 well plates at 10,000 cells per well in 3a or cytokine-containing medium for 21 days. Immunophenotypic analyses were performed using flow cytometry. A: Gating strategy for HSPCs and HSCs analyses. B-D: Cell number (left), and fold increase (middle), and percentage of all cells (right) tracked over 21 days in 3a versus cytokine medium. B: Live cells, C: HSPCs, D: HSCs. Pooled data from two donors. Mean ± SEM. **P < 0.01, ***P < 0.001, ****P < 0.0001 by 2-way ANOVA with Šídák's multiple comparisons test.

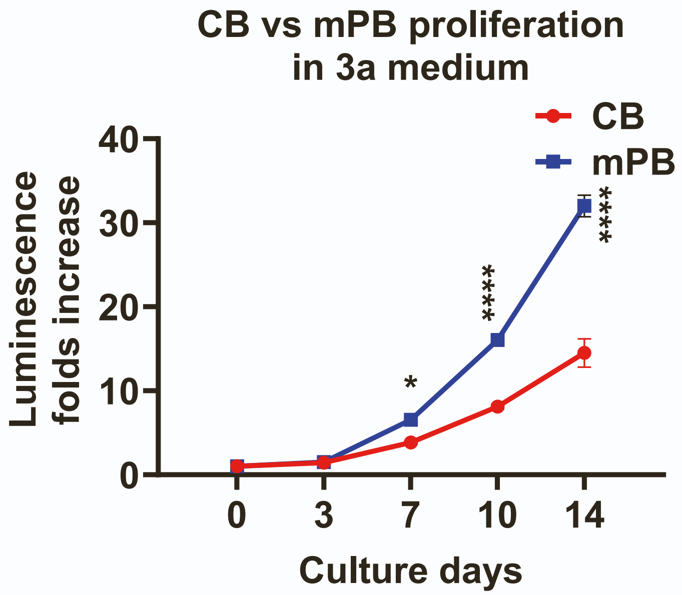

Figure S2: Cord blood vs peripheral blood cell proliferations in 3a medium. 25,000 human cord blood or peripheral blood-derived CD34+ cells were seeded in 96 well plate and cultured in 3a medium for 14 days. At each time point as indicated, cell proliferation was assessed using CellTiter-Glo® Luminescent Cell Viability Assay, data shows luminescence folds increase relative to the cells seeded at Day0. Data represents two times independent experiments. Data presented as mean ± SEM. *$P < 0.05$, ****$P < 0.0001$, by 2-way ANOVA with Šídák's multiple comparisons test.

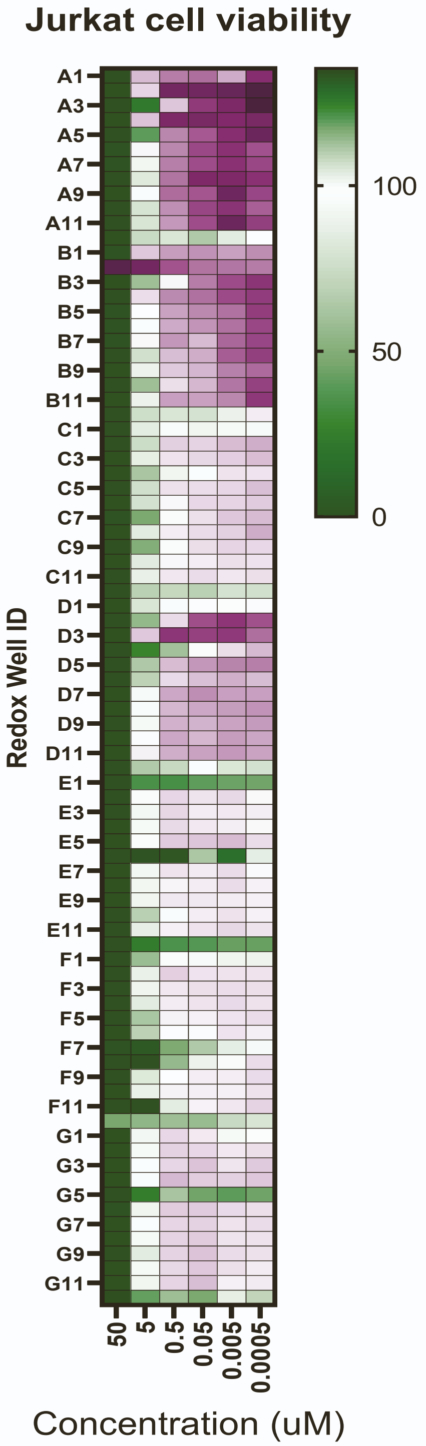

Figure S3: Titration of 83 antioxidant compound concentrations. Jurkat cells were seeded in 96 well plates at 10,000 cells per well in RPMI 1640 medium containing 1% Pen-Strep and 10% FBS. Cells were individually treated with 83 antioxidants at various concentrations ranged from 0.0005 to 50 uM. After 48 hours, cell proliferation was assessed using CellTiter-Glo® Luminescent cell viability assay, data shows luminescence folds relative to untreated cells. Data represents two times independent experiments.

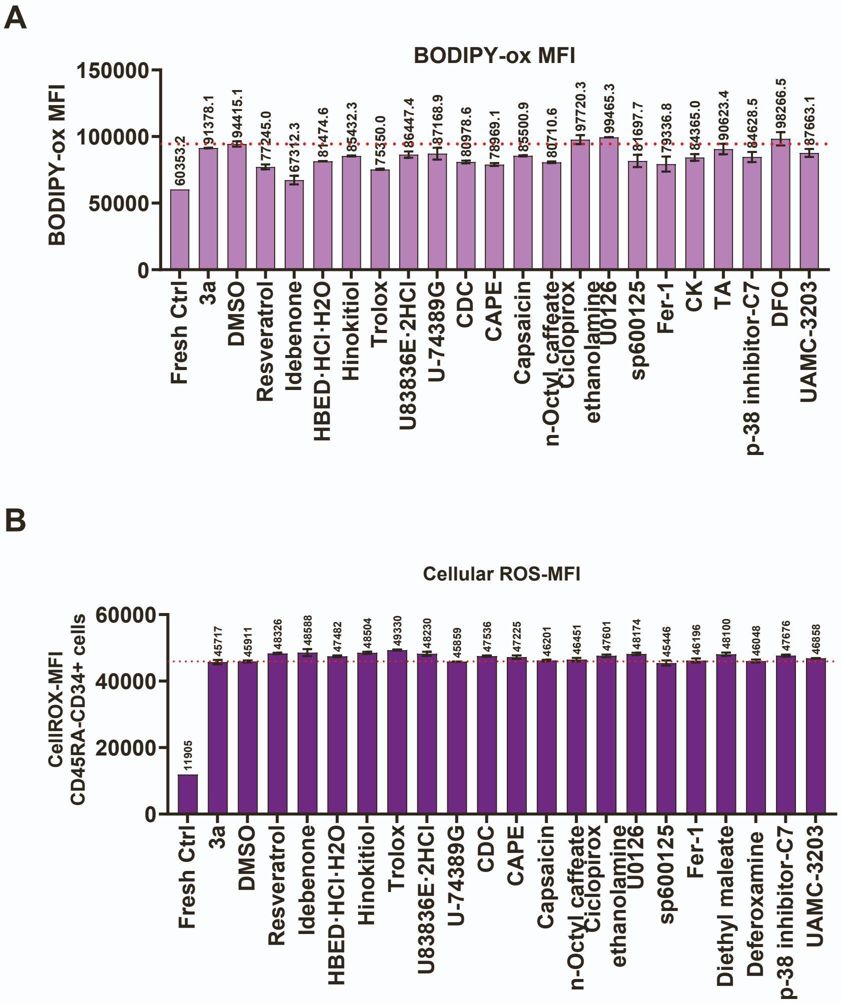

Figure S4: Lipid peroxidation and cellular ROS levels following treatment with selected compounds. Human CB cells were seeded in 96 well plates at 10,000 cells per well and cultured in 3a medium with selected compounds. A) Lipid peroxidation levels at day 14 analyzed by staining cells with BODIPY™ 581/591 C11. Data shows MFI in HSPCs. B) Cellular ROS levels at day 14 measured by staining cells with CellROX™ Deep Red. Data shows MFI in HSPCs. Representative data from three independent experiments are shown..

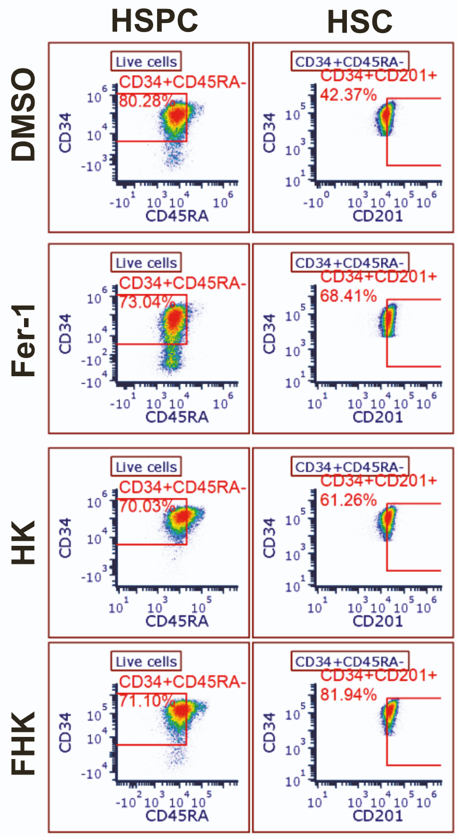

Figure S5: Representative FACS plots showing HSPC and HSC populations under each treatment conditions.

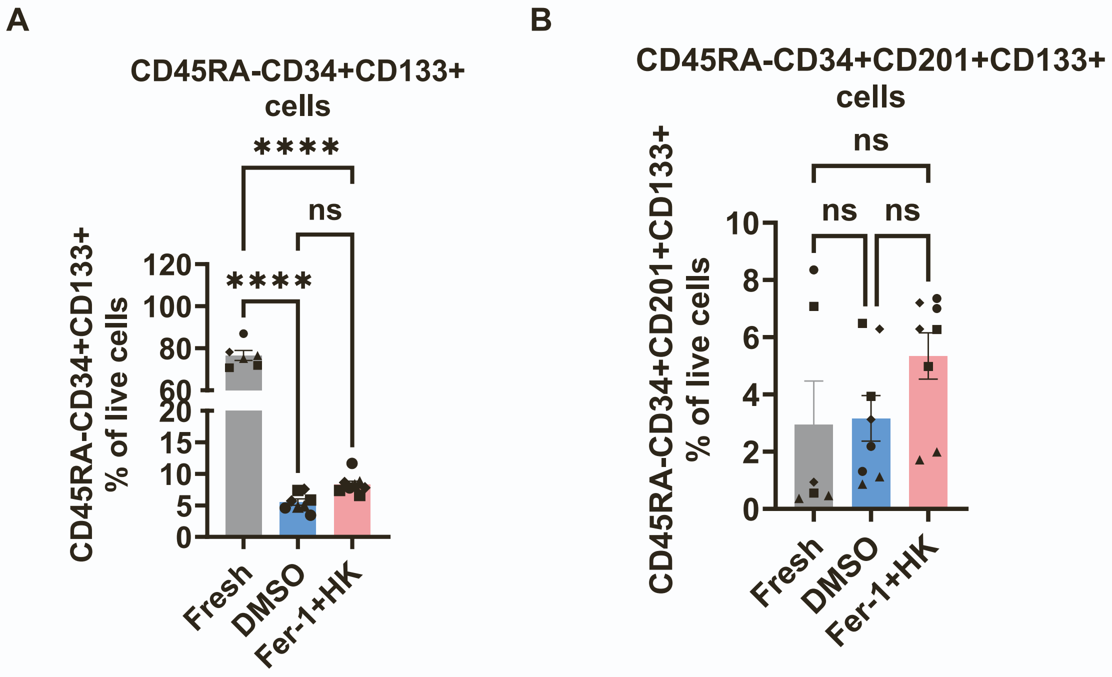

Figure S6: Immunophenotype analyses of *ex vivo* expanded CB cells in the presence of Fer-1 and Hinokitiol. Human CB cells were seeded in 6-well plates at 300,000 cells per well in 3a medium with DMSO (vehicle control), or Fer-1 (10uM) plus Hinokitiol (0.5uM) (FHK). A-B) Percentage of human CD45RA-CD34+CD133+ cells (A) and CD45RA-CD34+CD201+CD133+ (B) in live cells at day 14. Pooled data from three independent experiments with CD34+ cells from 4 donors (N=4) are presented as mean ± SEM. ****P < 0.0001 by 1-way ANOVA with Tukey's multiple-comparison test.

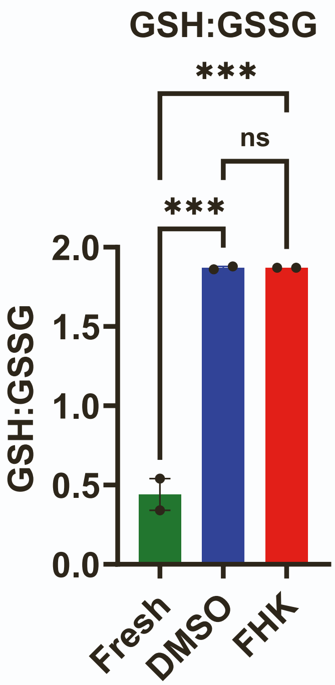

Figure S7: Effect of Hinokitiol and Fer-1 combination on human cord blood cell GSH:GSSG ratio. Human CB cells were cultured in 6-well plates at 300,000 cells per well and treated with DMSO (vehicle Ctrl), or Fer-1 plus Hinokitiol (FHK) for 14 days before GSH:GSSG ratio were measured following manufacture's instruction. Data presented as mean ± SEM by 1-way ANOVA with Tukey's multiple-comparison test. Pooled data from two donors are presented.

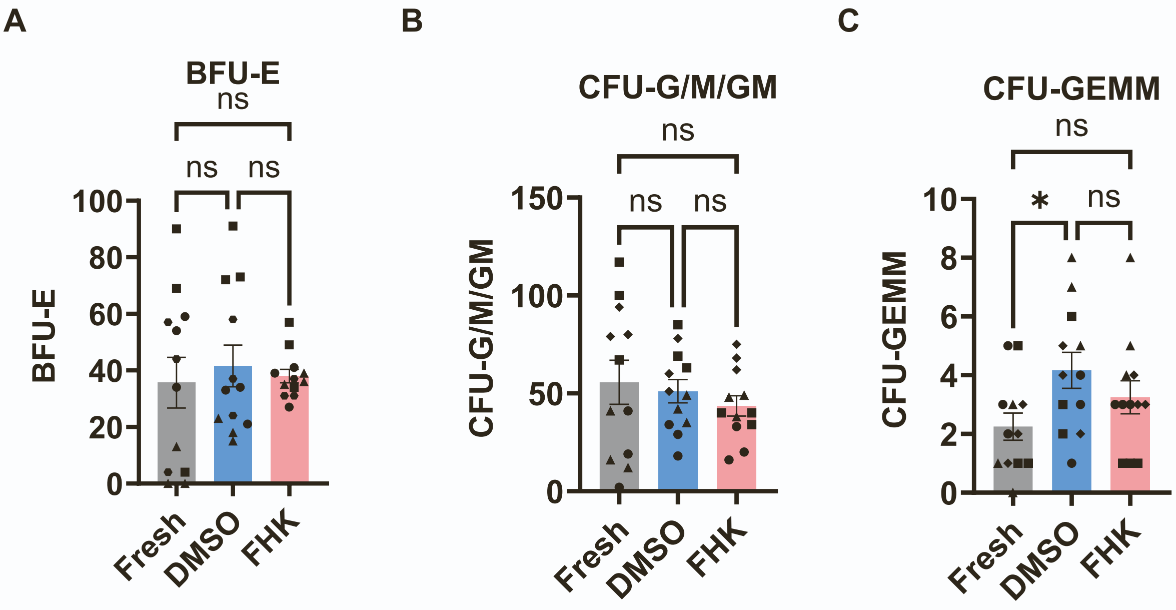

Figure S8: Effect of Hinokitiol and Fer-1 combination on human CB differentiation potential. Human CB cells were cultured in 6 well plates at 300,000 cells per well and treated with DMSO (vehicle Ctrl), or Fer-1 plus Hinokitiol (FHK) for 14 days. Then colony-forming unit (CFU) assay was initiated by seeding 1500 cultured cells per well in SmartDishTM 6-Well plates and culture at 37 °C with 5% CO2. After 14 days, plates were read with STEMvisionTM Automated CFU Assay Reader. Colony count of various types of colonies, including BFU-E (A), CFU-G/M/GM (B), and CFU-GEMN (C). Pooled data from three independent experiments with 4 donors showing colony count for various types. Data presented as mean ± SEM by 1-way ANOVA with Tukey's multiple-comparison test..

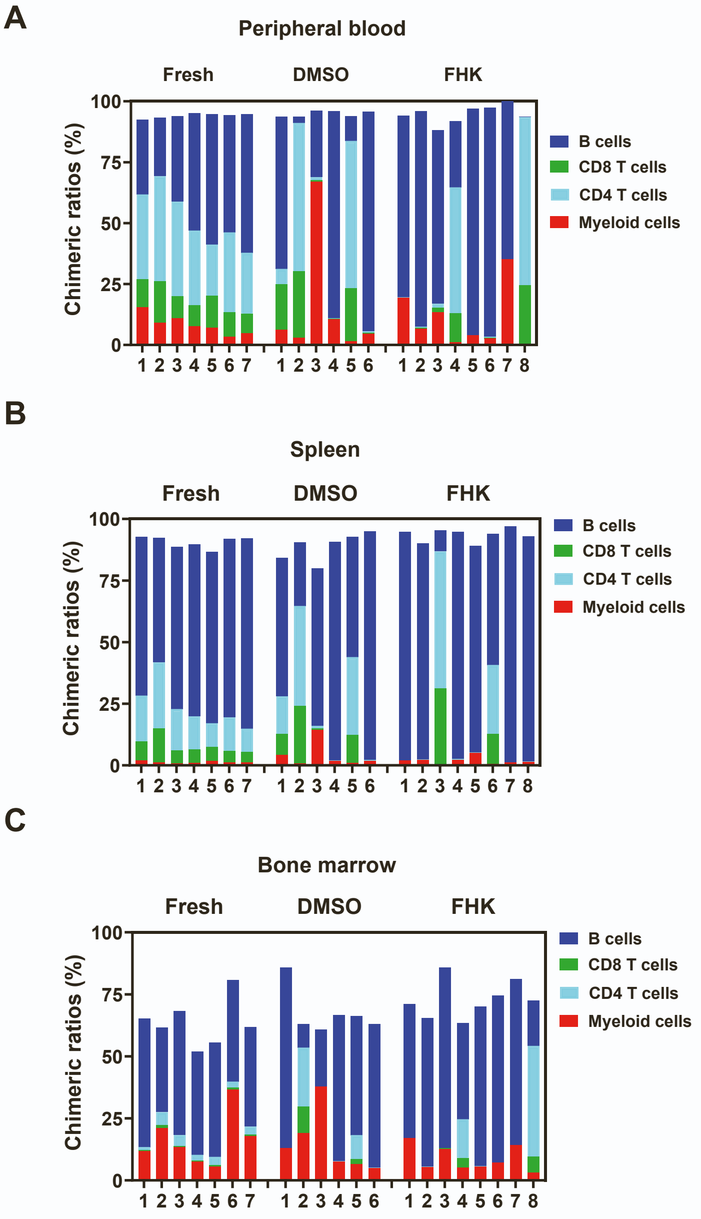

Figure S9: Lineage analyses of human blood cells in transplanted mice. Peripheral blood (A), spleen (B), and bone marrow (C) samples from individual mouse were analyzed at week 24 post-transplantation for lineage distribution of human blood cells by flow cytometry. X-axis showing number of mice in each group.

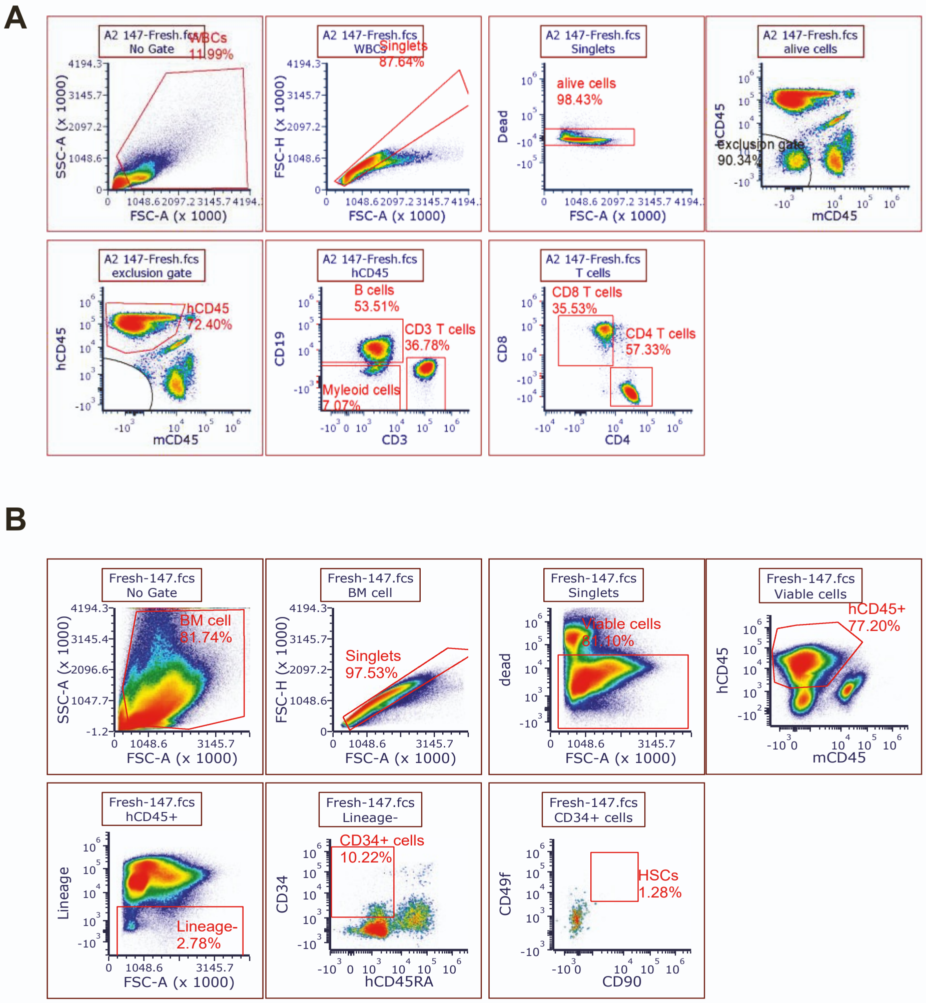

Figure S10: Representative FACS plots showing gating scheme for lineage (A) and HSPC (B) analyses.

Table S1: List of reagents.

Reagents	Clone	Flurophore	Vendor	Catalog
Alexa Fluor® 647 anti-human CD34	581	AF647	Biolegend	343508
BV421 Mouse Anti-Human CD34	581	BV421	BD Biosciences	562577
BV421 Mouse IgG1, k Isotype Control	X40	BV421	BD Biosciences	562438
Brilliant Violet 750™ anti-human CD45RA	HI100	BV750	Biolegend	304166
Brilliant Violet 750™ Mouse IgG1, κ isotype Ctrl	MOPC-21	BV750	Biolegend	400106
PE-labeled anti-human CD49c (ITGA3)	C3 II	PE	BD Biosciences	556025
APC anti-human CD133	clone 7	APC	Biolegend	372806
BV605 Rat Anti-Human CD201	RCR-252	BV605	BD Biosciences	743553
Brilliant Violet 421™ anti-human CD90 (Thy1)	5E10	BV421	Biolegend	328122
Brilliant Violet 785™ anti-human CD38	HIT2	BV785	Biolegend	303530
BV605 Rat IgG1, k Isotype Control	R3-34	BV605	BD Biosciences	562993
FITC Human lineage cocktail 4	--	FITC	BD Biosciences	562722
Zombie yellow viability kit.	---	---	Biolegend	423103
Zombie Aqua™ Fixable Viability Kit	---	---	Biolegend	423102
Human TruStain FcX™ (Fc Receptor Blocking Solution)	--	--	Biolegend	422302
Cell Staining Buffer	---	---	Biolegend	420201
Compensation Beads	---	---	Biolegend	424602

Table S2: List of compounds in Screen-Well REDOX Library (BML-2835 Version 1.4).

No.	Plate Location	Cat. No.	Compound	CAS #	MW	Conc.	Solvent	Activity
1	A-01	AC765	Promethazine·HCl	58-33-3	320.9	10mM	DMSO	Secondary anti-oxidant
2	A-02	RD100	Cumene hydroperoxide	80-15-9	152.2	10mM	DMSO	Aryl hydroperoxide
3	A-03	GR308	β-Lapachone	4707-32-8	242.3	10mM	DMSO	Undergoes futile redox cycles
4	A-04	FR104	Resveratrol	501-36-0	228.2	10mM	DMSO	Stilbene phenolic antioxidant
5	A-05	RD101	Hydroquinone	123-31-9	110.1	10mM	DMSO	Phenolic antioxidant
6	A-06	FR118	TEMPOL	2226-96-2	172.2	10mM	DMSO	SOD mimetic
7	A-07	FR116	Ferulic acid ethylester	4046-02-0	222.2	10mM	DMSO	Phenolic antioxidant
8	A-08	FR115	D-α-Tocopherylquinone	7559-04-8	446.7	10mM	DMSO	Oxidized vitamin E
9	A-09	RA119	Seratrodast	112665-43-7	354.4	10mM	DMSO	Quinone antioxidant
10	A-10	FR114	Idebenone	58186-27-9	338.4	10mM	DMSO	Quinone antioxidant
11	A-11	270061	t-Butylhydroquinone	1948-33-0	166.2	10 mM	DMSO	Phenolic antioxidant
12	A-12	FR113	HBED·HCl·H2O	35369-53-0 (base molecule)	442.9	10 mM	DMSO	Metal chelator
13	B-01	FR112	Ambroxol	18683-91-5	378.1	10mM	DMSO	Nonphenolic antioxidant
14	B-02	FR111	L-Ergothioneine	497-30-3	229.3	10mM	H2O	Endogenous antioxidant
15	B-03	FR110	Hinokitiol	499-44-5	164.2	10mM	DMSO	Metal chelator
16	B-04	270263	(-)-Epigallocatechin gallate	989-51-5	458.4	10 mM	DMSO	Polyphenol
17	B-05	FR106	Procysteine	19771-63-2	147.2	10mM	DMSO	Glutathione precursor
18	B-06	FR105	Trolox	53188-07-1	250.3	10mM	DMSO	Short-chain vitamin E analog
19	B-07	FR103	MCI-186	89-25-8	174.2	10mM	DMSO	Nonphenolic antioxidant
20	B-08	FR101	U83836E·2HCl	137018-55-4	593.6	10mM	DMSO	Antioxidant Lazaroid
21	B-09	FR100	U-74389G	153190-29-5	726.9	10 mM	DMSO	Antioxidant Lazaroid
22	B-10	EI363	GERI-BP002A	119-47-1	340.5	10mM	DMSO	Phenolic antioxidant
23	B-11	EI345	Apigenin	520-36-5	270.2	10mM	DMSO	Flavone antioxidant
24	B-12	EI318	Terbinafine·HCl	78628-80-5	327.9	10mM	DMSO	Free radical quencher
25	C-01	270253	Rosmarinic acid	537-15-5, 20283-92-5	360.3	10 mM	DMSO	Phenolic antioxidant
26	C-02	270202	Piceatannol	10083-24-6	244.2	10 mM	DMSO	Stilbene phenolic antioxidant
27	C-03		Blank					

28	C-04	EI211	CDC	132465-11-3	321.3	10mM	DMSO		Phenolic antioxidant
29	C-05	EI207	Ebselen	60940-34-3	274.2	10mM	DMSO		Glutathione peroxidase mimetic
30	C-06	350006	Genistein	446-72-0	270.2	10mM	DMSO		Isoflavone antioxidant
31	C-07	EI135	Curcumin	458-37-7	368.4	10mM	DMSO		Phenolic antioxidant
32	C-08	EI134	Phenidone	92-43-3	162.2	10mM	DMSO		Nonphenolic antioxidant
33	C-09	EI130	Gossypol	303-45-7	518.6	10mM	DMSO		Phenolic antioxidant
34	C-10	EI129	Gentisic acid	490-79-9	154.1	10mM	DMSO		Phenolic antioxidant
35	C-11	EI124	Caffeic acid	331-39-5	180.2	10mM	DMSO		Phenolic antioxidant
36	C-12	EI106	Baicalein	491-67-8	270.2	10mM	DMSO		Flavone antioxidant
37	D-01	EI100	Esculetin	305-01-1	178.1	10mM	DMSO		Coumarin antioxidant
38	D-02	RD103	Propyl gallate	121-79-9	212.2	10mM	DMSO		Phenolic antioxidant
39	D-03	ET004	5,8,11,14-Eicosatetraynoic acid	1191-85-1	296.4	10mM	DMSO		Acetylenic antioxidant
40	D-04	FR102	CAPE	104594-70-9	284.3	10mM	DMSO		Phenolic antioxidant
41	D-05	EI101	Nordihydroguaiaretic acid	500-38-9	302.4	10mM	DMSO		Phenolic antioxidant
42	D-06	EI125	Capsaicin	404-86-4	305.4	10mM	DMSO		Phenolic antioxidant
43	D-07	RD104	BHT	128-37-0	220.4	10mM	DMSO		Phenolic antioxidant
44	D-08	RD105	BHA	121-00-6	180.2	10mM	DMSO		Phenolic antioxidant
45	D-09	350144	Bakuchiol	10309-37-2	256.4	10mM	DMSO		Phenolic antioxidant
46	D-10	RD106	DL-α-Lipoic acid	1077-28-7	206.3	10mM	DMSO		Sulfur-containing antioxidant
47	D-11	RD107	Eugenol	97-53-0	164.2	10mM	DMSO		Phenolic antioxidant
48	D-12	NS520	Melatonin	73-31-4	232.3	10mM	DMSO		Nonphenolic antioxidant
49	E-01	105005	N-Acetyl-Cysteine	616-91-1	163.2	10mM	DMSO		Thiol-containing reducing agent
50	E-02	RD109	D-γ-Tocopherol	54-28-4	416.7	10mM	DMSO		Phenolic antioxidant
51	E-03	AP308	Tocopherol succinate	4345-03-3	530.8	10mM	DMSO		Phenolic antioxidant
52	E-04	RD110	Ascorbic acid	50-81-7	176.1	10mM	DMSO		Ascorbate-type antioxidant
53	E-05	RD111	Ascorbyl palmitate	137-66-6	414.5	10mM	DMSO		Lipohilic ascorbate
54	E-06	RD112	n-Octyl caffeate	NA	292.4	10mM	DMSO		Phenolic antioxidant
55	E-07	RD113	Paeonol	552-41-0	166.2	10mM	DMSO		Phenolic antioxidant
56	E-08	RD129	Protocatechuic acid	99-50-3	154.1	10mM	DMSO		Phenolic antioxidant
57	E-09	RD114	Glutathione	70-18-8	307.3	10mM	DMSO		Thiol-containing reducing agent

58	E-10	AR112	Carvedilol	72956-09-3	406.5	10mM	DMSO	Nonphenolic antioxidant
59	E-11	RD115	Diludine	1149-23-1	253.3	10 mM	DMSO	Dihydropyridine-type antioxidant
60	E-12	270264	Carnosic acid	3650-09-7	332.4	10mM	DMSO	Phenolic antioxidant
61	F-01	GR336	Tanshinone IIA	568-72-9	294.3	10mM	DMSO	1,2-Quinone antioxidant
62	F-02	RD116	Probucol	23288-49-5	516.8	10mM	DMSO	Phenolic antioxidant
63	F-03	FA001	Eicosapentaenoic acid	10417-94-4	302.5	10 mM	DMSO	Polyunsaturated radical scavenger
64	F-04	FA002	Docosa-4Z,7Z,10Z,13Z,16Z,19Z-hexaenoic acid	6217-54-5	328.5	10mM	DMSO	Polyunsaturated radical scavenger
65	F-05	NP576	Bis demethoxycurcumin	24939-16-0	308.3	10 mM	DMSO	Phenolic antioxidant
66	F-06	EI375	Ibuproxam	53648-05-8	221.3	10mM	DMSO	Metal chelator
67	F-07	RD128	Ciclopirox ethanolamine	41621-49-2	268.4	10mM	DMSO	Hydroxyl radical scavenger
68	F-08	NP536	Thymoquinone	490-91-5	164.2	10mM	DMSO	Quinone antioxidant
69	F-09	RD117	Thiourea	62-56-6	76.1	10mM	DMSO	Thiol-containing reducing agent
70	F-10	280001	DTT	3483-12-3	154.3	10 mM	DMSO	Thiol-containing reducing agent
71	F-11	PI149	N-Ethylmaleimide	128-53-0	125.1	10mM	DMSO	Thiol trap
72	F-12	FR117	L-Buthionine-sulfoximine	83730-53-4	222.3	10 mM	water	Glutathione biosynthesis inhibitor
73	G-01	DL151	Anethole trithione	532-11-6	240.4	10mM	DMSO	Sulfur-containing antioxidant
74	G-02	RD119	TEMPO	2564-83-2	156.2	10mM	DMSO	Nitroxyl radical
75	G-03	ST330	D609	83373-60-8	266.5	10mM	DMSO	Antioxidant
76	G-04	270212	Captopril	62571-86-2	217.3	10 mM	DMSO	Thiol-containing reducing agent
77	G-05	DL379	Disulfiram	97-77-8	296.5	10 mM	DMSO	Sulfur-containing antioxidant
78	G-06	RD121	1,2-Dithiole-3-thione	534-25-8	134.2	10 mM	DMSO	Sulfur-containing antioxidant
79	G-07	RD122	Selenomethionine	3211-76-5	196.1	10mM	DMSO	Selenium-containing antioxidant
80	G-08	RD123	Tetramethylpyrazine	1124-11-4	136.2	10mM	DMSO	Nonphenolic antioxidant
81	G-09	RD124	Ethoxyquin	91-53-2	217.3	10mM	DMSO	Nonphenolic antioxidant
82	G-10	RD125	Canthaxanthin	514-78-3	564.8	1mM	DMSO	Polyunsaturated radical scavenger
83	G-11	RD126	β-carotene	7235-40-7	536.9	10mM	DMSO	Polyunsaturated radical scavenger
84	G-12	RD127	Retinyl palmitate	79-81-2	524.9	10mM	DMSO	Polyunsaturated radical scavenger
